# MG1 interacts with a protease inhibitor and confers resistance to rice root-knot nematode

**DOI:** 10.1038/s41467-023-39080-6

**Published:** 2023-06-08

**Authors:** Xiaomin Wang, Rui Cheng, Daochao Xu, Renliang Huang, Haoxing Li, Liang Jin, Yufeng Wu, Jiuyou Tang, Changhui Sun, Deliang Peng, Chengcai Chu, Xiaoli Guo

**Affiliations:** 1 State Key Laboratory of Agricultural Microbiology, College of Plant Science and Technology, Huazhong Agricultural University, Wuhan, 430070 China; 2 Nanchang Subcenter of Rice National Engineering Laboratory, Key Laboratory of Rice Physiology and Genetics of Jiangxi Province, Rice Research Institute, Jiangxi Academy of Agricultural Sciences, Nanchang, 330200 China; 3 State Key Laboratory of Agricultural Microbiology, College of Life Science and Technology, Huazhong Agricultural University, Wuhan, 430070 China; 4 State Key Laboratory for Crop Genetics and Germplasm Enhancement, Bioinformatics Center, Academy for Advanced Interdisciplinary Studies, Nanjing Agricultural University, Nanjing, 210095 China; 5 State Key Laboratory of Plant Genomics, National Center for Plant Gene Research (Beijing), Institute of Genetics and Developmental Biology, Chinese Academy of Sciences, Beijing, 100101 China; 6 State Key Laboratory of Crop Gene Exploration and Utilization in Southwest China, Rice Research Institute, Sichuan Agricultural University, Chengdu, 625014 China; 7 State Key Laboratory for Biology of Plant Diseases and Insect Pests, Institute of Plant Protection, Chinese Academy of Agricultural Sciences, Beijing, 100193 China

**Keywords:** Plant immunity, Plant genetics, Plant signalling

## Abstract

The rice root-knot nematode (*Meloidogyne graminicola*) is one of the most destructive pests threatening rice (*Oryza sativa* L.) production in Asia; however, no rice resistance genes have been cloned. Here, we demonstrate that *M. GRAMINICOLA-RESISTANCE GENE 1* (*MG1*), an *R* gene highly expressed at the site of nematode invasion, determines resistance against the nematode in several rice varieties. Introgressing *MG1* into susceptible varieties increases resistance comparable to resistant varieties, for which the leucine-rich repeat domain is critical for recognizing root-knot nematode invasion. We also report transcriptome and cytological changes that are correlated with a rapid and robust response during the incompatible interaction that occurs in resistant rice upon nematode invasion. Furthermore, we identified a putative protease inhibitor that directly interacts with MG1 during *MG1*-mediated resistance. Our findings provide insight into the molecular basis of nematode resistance as well as valuable resources for developing rice varieties with improved nematode resistance.

## Abstract (web summary)

Rice root-knot nematode is a major threat to rice production. Here, the authors identify an *R* gene *MG1* in rice that confers resistance against nematodes and encodes a protein that interacts with a protease inhibitor.

## Introduction

Rice (*Oryza sativa* L.) is a major staple crop, providing food for more than half of the world’s population^1^. Plant-parasitic nematodes cause 10–25% annual yield losses in rice worldwide^2^. Over 100 different nematode species attack rice^3^. Among them, root-knot nematodes (*Meloidogyne* spp.), one of the top 10 economically important plant-parasitic nematodes, have a remarkably wide host range and cause the most damage^4^. Rice root-knot nematode (*M. graminicola*) is the most prevalent root-knot nematode in rice cultivation systems and is a major threat to rice production, particularly in Asia, where ~90% of the world’s rice is produced and consumed^5–7^.

*M. graminicola* is an obligate sedentary endoparasite that is widely distributed in South East Asia and other countries where rice is extensively cultivated (Mg distribution map: https://www.cabidigitallibrary.org/doi/10.1079/cabicompendium.33243)^2,8^. *M. graminicola* infects upland, lowland, and deep-water rice, as well as rice grown in nurseries. Field populations of rice root-knot nematode are increasing dramatically due to changes in agricultural practices in response to water shortage and climate change, such as the development of aerobic rice^5^.

The infective second-stage juvenile (J2) of rice root-knot nematode enters the root in the elongation zone and migrates toward the vasculature, where it stimulates rice tissues to form a specialized feeding structure called giant cells, which provide the needed nourishment to complete their life cycle^6,9^. After feeding, the J2s molt three more times to reach the reproductive adult stage, and the life cycle is completed within 2–3 weeks depending on the temperature and flooding conditions in the fields^10^. Unlike the symptoms caused by other *Meloidogyne* species, *M. graminicola* induces hyperplasia and hypertrophy of surrounding rice root cells, resulting in hook-shaped galls at the root tips (Supplementary Fig. 1a). The adult females lay eggs inside the galls, where they are well-protected from flooded conditions, and J2s of the next generation will continue the infection cycle^11^. Consequently, substantial damage to the rice root system leads to poor growth (Supplementary Fig. 1b) and severe rice yield losses^7^.

Control strategies such as crop rotation, continuous flooding, and nematicides, which are commonly applied in the field to mitigate yield losses caused by *M. graminicola*, are not efficient or feasible^7^. Breeding more resistant varieties offers an effective, economical, and environmentally friendly option to manage these nematodes. For example, one nematode resistance gene, *Mi-1*.*2*, found in the wild tomato species *Lycopersicon peruvianum*, is used commercially to enhance resistance to several root-knot nematode species in cultivated tomato (*Solanum lycopersicum*)^12,13^. *Ma* from Myrobalan plum (*Prunus cerasifera*) confers broad-spectrum resistance to over 30 root-knot nematode species^14^ and is utilized for rootstock breeding in stone fruits^15^.

Rice germplasm constitutes natural sources of resistance against *M. graminicola*. Resistance to this nematode has been found in two rice species from Africa, the wild rice *Oryza longistaminata* and domesticated *O. glaberrima*^16^. However, introgressing resistance genes into Asian rice (*O. sativa*) is challenging because the interspecific progeny fails to express the same degree of resistance observed in African rice^17^. A new source of resistance has been identified recently in another wild rice species, *O. glumaepatuma*^18^. It has been reported that multiple quantitative trait loci (QTLs) are responsible for *M. graminicola* resistance traits using different mapping populations, but all of these loci need to be clarified. Six QTLs controlling partial nematode resistance were located on chromosomes 1, 2, 6, 7, 9, and 11 using recombinant inbred lines (RILs) derived from a cross between the *O. sativa* varieties Bala and Azucena^19^. Jena et al.^20^ reported two QTLs on chromosomes 1 and 3 from the analysis of RILs derived from a cross between the varieties Annapurna and Ramakrishna. Likewise, more QTLs associated with *M. graminicola* resistance and tolerance have been detected in populations derived from a cross between the aerobic rice genotype IR78877–208-B-1-2 and the *O. glaberrima* genotype CG14^21,22^. In addition, a genome-wide association study (GWAS) of a global rice panel revealed 11 genomic regions associated with nematode resistance, distributed on chromosomes 1, 3, 4, 5, 11, and 12^23^. Similarly, 17 single nucleotide polymorphisms (SNPs) positively associated with nematode resistance were identified in Indian wild rice accessions via GWAS^24^.

Although most Asian rice germplasm are susceptible to *M. graminicola*, a few highly resistant varieties have been reported in Asian rice, such as LD24 (an *indica* rice from Sri Lanka), Khao Pahk Maw (KPM, an *aus* rice from Thailand), and Zhonghua 11 (ZH11, a *japonica* rice from China)^23,25^. Recent studies suggested that *M. graminicola* resistance is governed by a major locus, and the trait can be qualitative in certain varieties. In Asian rice (cv. Abhishek), a resistance locus was mapped to chromosome 10 through bulk segregant analysis^26^. Phan et al.^25^ reported that a potential dominant gene(s) confers incompatible nematode resistance in ZH11 with a hypersensitivity-like reaction (HR) following nematode infection. QTL sequencing (QTL-seq) of two rice populations generated from crosses between the resistant varieties LD24 or KPM and the susceptible variety Vialone Nano identified the same locus on chromosome 11, which was responsible for the resistance in LD24 or KPM^27^. Despite these efforts, no candidate gene conferring complete resistance for breeding resistant rice cultivars has been cloned.

Here, we screened 207 Asian rice (*O. sativa*) varieties and identified three novel sources (SL 22-620, HKG 98, and Toga), along with the previously reported variety ZH11, as being fully resistant to *M. graminicola* infection. One allelic dominant gene, *M. GRAMINICOLA-RESISTANCE GENE 1* (*MG1*), determined the nematode resistance derived from SL 22-620, HKG 98, and ZH11. Map-based cloning and functional analysis revealed that *MG1* encodes a coiled-coil, nucleotide-binding, and leucine-rich repeat (CC-NB-LRR) protein, whose LRR domain plays a prominent role in its nematode resistance activity. Further, we identified a putative protease inhibitor involved in MG1-mediated defense responses. MG1-mediated resistance should protect rice from *M. graminicola*, offering a completely dominant trait for rice root-knot nematode resistance breeding.

## Results

### Screening rice resistance to *M. graminicola*

**Fig. 1:**
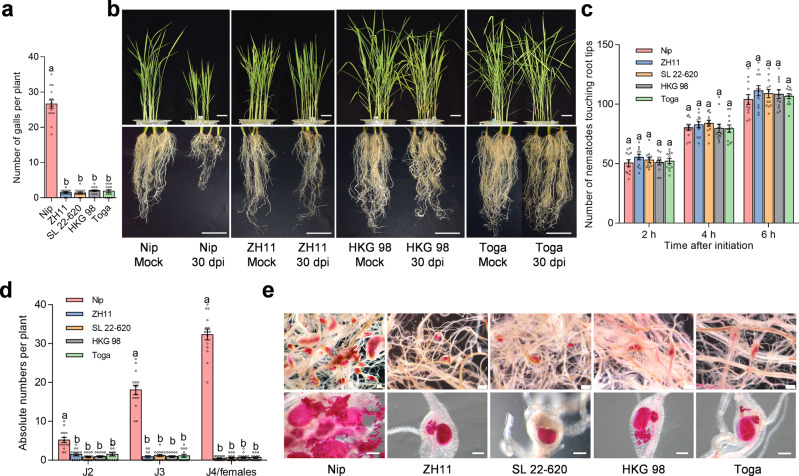
Comparison of resistance phenotypes in different rice varieties against *M. graminicola* infection. **a** Number of galls on resistant (ZH11, SL 22-620, HKG98, and Toga) and susceptible (Nipponbare) rice varieties at 15 dpi with *M. graminicola*. Fourteen-day-old rice plants were inoculated with 150 J2s. Data are means ± standard error of mean (s.e.m.) (*n* = 15 independent plants). **b** Morphology of nematode-infected plants at 30 dpi. **c** Nematode attraction assay of different rice varieties. Root tips were placed in a 12-well plate containing 1000 J2s; the number of J2s touching the root tips was counted at 2, 4, and 6 hpi. Data represent means ± s.e.m. (*n* = 12 independent plants). **d** Number of nematodes at the J2, J3, and J4/female developmental stages in different rice varieties at 15 dpi. Data are means ± s.e.m. (*n* = 15 independent plants). J2, second-stage juvenile; J3, third-stage juvenile; J4, fourth-stage juvenile. **e** Acid fuchsin staining of rice roots at 15 dpi. Lower panel shows representative images of one gall from the upper panel. Scale bars, 5 cm (**b**), 500 μm (**e**). Different letters in (**a**, **c**, **d**) above the bars indicate statistical significance groups at *P* < 0.05 (one-way ANOVA analysis followed by Fisher’s LSD multiple comparison test). Exact *P* values are provided in the Source Data file. All experiments were performed three times with similar results.

To identify resistant sources against *M. graminicola*, we evaluated 207 rice varieties, comprising 197 lines of the United States Department of Agriculture (USDA) Rice Mini-Core Collection^28^ representing the genetic diversity found in 18,709 rice varieties collected worldwide and 10 lines collected in our laboratory, by assessing gall numbers in nematode-infected plants under controlled conditions. Most rice varieties were susceptible to nematode infection, with substantial variation observed across varieties 2 weeks after inoculation (Supplementary Fig. 2, Supplementary Table 1). However, we identified three resistant varieties, Toga (MC79, an *indica* rice from India), SL 22-620 (MC162, an *aus* rice from Sierra Leone), and HKG 98 (MC174, an *aus* rice from Mali), and the previously identified resistant *japonica* variety ZH11. Of the four varieties, Toga exhibited the lowest gall number. Plants from these resistant varieties bore at most two galls, compared to 27 galls in the susceptible *japonica* variety Nipponbare (Fig. 1a). Plant growth was barely affected in the resistant varieties after *M. graminicola* infection, in contrast to the stunted growth observed for Nipponbare (Fig. 1b). We performed a nematode attraction assay by counting the number of J2s touching the root tip at 2, 4, and 6 h after inoculation and observed no significant difference between Nipponbare and the four resistant varieties (Fig. 1c, Supplementary Fig. 3). We also assessed nematode penetration and development after inoculation. In the root system of the resistant rice varieties, the number of nematodes was significantly lower and nematode development was delayed compared to Nipponbare (Fig. 1d, e; Supplementary Fig. 4). At 15 days post inoculation (dpi), Nipponbare galls were filled with eggs and females, whereas the resistant varieties contained few females or eggs.

### Molecular cloning of *MG1*

To investigate whether the nematode resistance of ZH11, Toga, SL 22-620, and HKG 98 is governed by the same locus, we crossed the resistant varieties to obtain a set of progeny for an allelism test. All F_1_ and F_2_ offspring derived from SL 22-620 × ZH11 and HKG 98 × ZH11 crosses exhibited the same resistant phenotype as their parents (Supplementary Table 2). However, the F_1_ plants derived from ZH11 × Toga and HKG 98 × Toga crosses were resistant, but the F_2_ offspring segregated for resistant:susceptible phenotypes in a 15:1 ratio (160:16 or 182:15; *χ2* = 2.42 or 0.63 < 3.84, *P* > 0.05) (Supplementary Table 2). This ratio indicated that the genes responsible for the resistance of SL 22-620, HKG 98, and ZH11 are allelic, while Toga carries a non-allelic resistance locus.

To map the common gene governing nematode resistance in SL 22-620, HKG 98, and ZH11, we developed three F_2_ populations segregating for nematode resistance using two resistant varieties, HKG 98 and ZH11, and three susceptible varieties, Nipponbare, MH63, and Lehui 188, as parents. The F_1_ plants derived from the crosses ZH11 × MH63, ZH11 × Lehui 188 and HKG 98 × Nipponbare were highly resistant to *M. graminicola* (Supplementary Fig. 5a–c). Gall number formed on individual plants for each F_2_ population showed a continuous distribution with an apparent valley in the distribution curve (Supplementary Fig. 5d–f), and the segregation of the resistant to susceptible plants fitted a 3:1 ratio (248:85, 249:75, or 155:36; *χ2* = 0.036, 0.498, or 3.534 < 3.84, *P* > 0.05) (Supplementary Fig. 5g). These results suggested that a single dominant gene, hereafter designated as *M. GRAMINICOLA-RESISTANCE GENE 1* (*MG1*), likely confers the nematode resistance observed in HKG 98 and ZH11.

Next, we carried out a bulk segregant analysis (BSA) using bulked resistant (R) and susceptible (S) F_2_ plants. We identified two polymorphic markers (11–20 M and 11–25 M) and three polymorphic markers (CR2, CR8, and FJ5) from a contiguous region on chromosome 11 that distinguish the R and S pools from the ZH11 × Lehui 188 and HKG 98 × Nipponbare populations, respectively (Supplementary Fig. 5h, i). We also used the bulked DNA samples for QTL-seq analysis, which highlighted a genomic region on chromosome 11 from 20.39 to 29.02 Mb with the most significant QTL peak (Supplementary Fig. 5j).

**Fig. 2:**
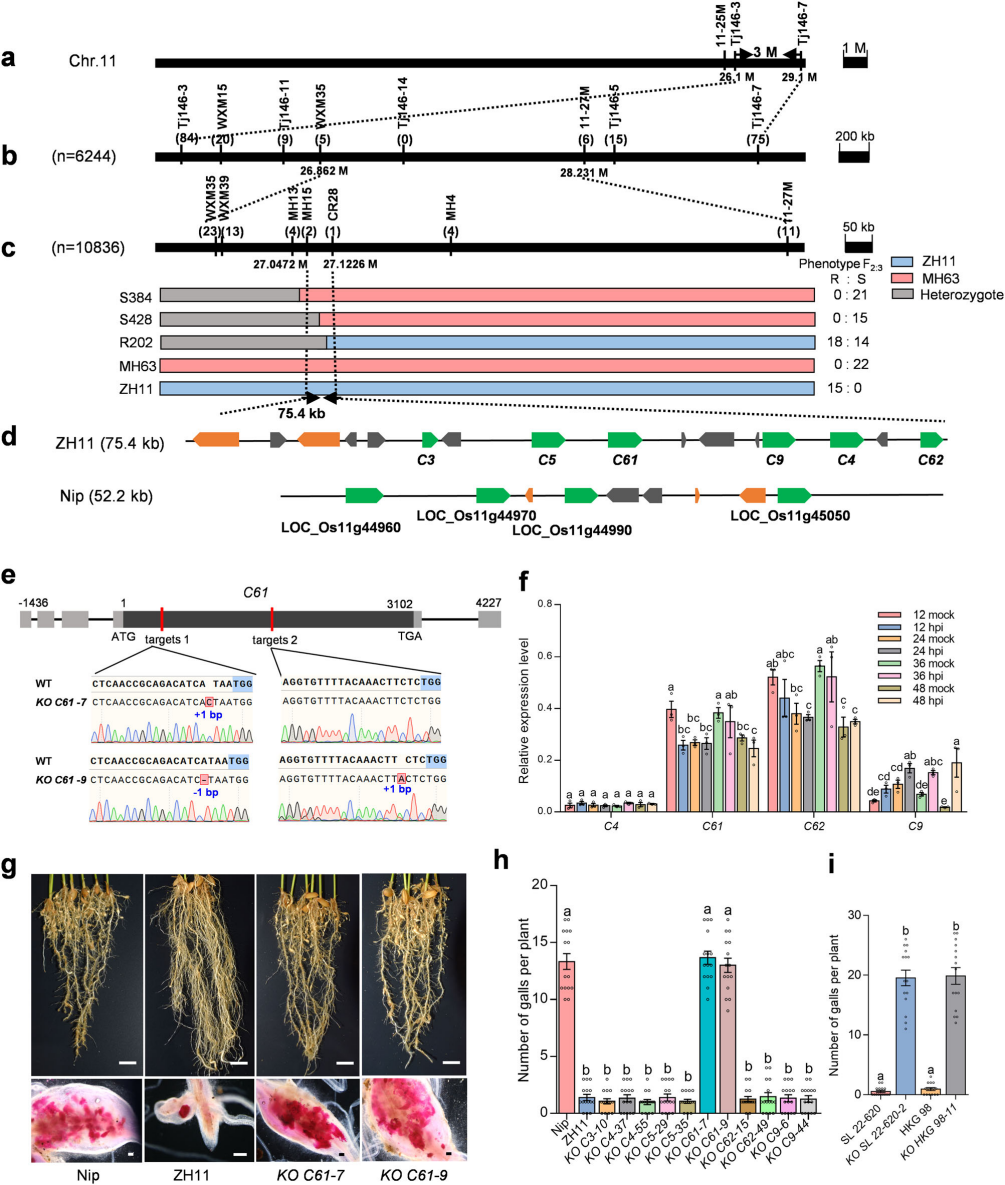
Map-based cloning of *MG1*. **a** *MG1* is located on the long arm of chromosome 11 between markers Tj146-3 and Tj146-7. **b**, **c** Fine-mapping of *MG1* to a 75.4-kb region flanked by markers MH15 and CR28 in ZH11. The number of recombinants detected between the molecular markers is indicated in parentheses below the markers. The numbers below the linkage map represent the location of the markers. Genotypes and S or R phenotypes of three recombinants are included in (**c**). Different color boxes denote the marker genotypes. **d** Predicted open reading frames within the mapping intervals in ZH11 and Nipponbare. Green arrows indicate the predicted *NLR*s, and orange arrows represent expressed genes. The transposable elements are indicated in gray. **e** Schematic diagram of *C61* showing the gene structure and two target sites. The coding region is shown in gray, and untranslated regions are in light gray. The lines represent introns. Sequences of target sites in the wild type and two homozygous mutants (*C61-7* and *C61-9*) are aligned below the gene structure. The protospacer adjacent motif (PAM) is marked with blue background. Blue numbers indicate deleted (−) or inserted (+) bases in two target sites. Insertions and deletions are highlighted with red background in the aligned sequences. Short lines indicate deletions. **f** Relative expression levels of the candidate genes in rice root tips at the indicated time points post infection with *M. graminicola*. Six-day-old rice seedlings inoculated with 150 J2s were subjected to RT-qPCR analysis using *OsEXPNAR* as an internal control. Data are means ± s.e.m. of three independent biological replicates with two technical replicates. **g** Representative images of *C61-*knockout mutants in the ZH11 background at 15 days after *M. graminicola* infection. **h** Number of galls on CRISPR-edited mutants in the ZH11 background at 15 dpi. Data are means ± s.e.m. (*n* = 15 independent plants). **i** Number of galls on *C61* mutants in the SL 22-620 and HKG 98 backgrounds. Data are means ± s.e.m. (*n* = 15 independent plants). Different letters above the bars in (**f**, **h**, **i**) indicate statistical significance groups at *P* < 0.05 (one-way ANOVA analysis followed by Fisher’s LSD multiple comparison test). Exact *P* values are provided in the Source Data file. Scale bars in (**g**), 2 cm (upper panel), 500 μm (lower panel). The experiments (**g**–**i**) were performed three times with similar results.

Using 6244 F_2_ plants derived from the cross between ZH11 and Lehui 188, we delimited *MG1* to a 26.86–28.23-Mb interval flanked by markers WXM35 and 11-27 M (Fig. 2a, b). High-resolution mapping using an additional 10,836 F_2_ and F_2:3_ plants derived from ZH11 × Lehui 188 (or MH63) narrowed the candidate region down to 52.2 kb between markers MH15 and CR28, based on the Nipponbare reference genome (Fig. 2c). In the ZH11 reference genome, the corresponding interval flanked by markers MH15 and CR28 covered 75.4 kb (Fig. 2d). Likewise, using 7929 F_2_ and F_2:3_ individuals derived from the cross between HKG 98 and Nipponbare, we further delimited *MG1* to a 38.3-kb interval between markers CR20 and CR28 in Nipponbare, which further narrowed the target region to 47.5 kb in ZH11 (Supplementary Fig. 6).

Sequence analysis located *MG1* within a gene cluster encoding nucleotide-binding (NB) and leucine-rich repeat (LRR) receptors (NLRs). When we compared the genome sequences of ZH11 and Nipponbare, we identified a large structural variation within our target region (Fig. 2d). The 52.2-kb region of Nipponbare contained four annotated NLR-like genes (LOC_Os11g44960, LOC_Os11g44970, LOC_Os11g44990, and LOC_Os11g45050), three genes encoding expressed proteins, and two retrotransposon genes. We preliminarily annotated the corresponding 75.4-kb region of ZH11 using open reading frame (ORF) (http://hollywood.mit.edu/GENSCAN.html) and protein structure (http://smart.embl-heidelberg.de/) predictions. Among the 16 genes predicted in this region, six genes encoded NLR-like proteins, which we designated *C3*, *C4*, *C5*, *C61*, *C62*, and *C9*; the remaining 10 genes were predicted to encode transposable elements or expressed proteins. Because NLR-like proteins are implicated in disease resistance, we selected the six NLR genes for further investigation. C3 is a truncated NLR protein with only the LRR domain, but was located outside of the 47.5-kb target interval deduced from mapping results of the HKG 98×Nipponbare population (Supplementary Fig. 6). Therefore, we considered *C4*, *C5*, *C61*, *C62*, and *C9* as the most likely candidates for *MG1*.

We obtained full-length cDNAs for each gene by rapid amplification of cDNA ends (RACE)-PCR (Fig. 2e, Supplementary Fig. 7a). None of the five remaining NLR genes contained introns in their coding region, and only the untranslated region (UTR) of *C61* contained four introns (Fig. 2e). Next, we performed reverse transcription quantitative PCR (RT-qPCR) to determine if the expression of these genes is induced by nematode infection. Only *C9* showed a nematode-induced expression pattern at 24–48 h post inoculation (hpi) (Fig. 2f). By contrast, *C61* expression did not significantly change from 24 to 48 hpi, but decreased at 3 and 4 dpi (Supplementary Fig. 7b). *C3* and *C5* had the lowest expression of all tested genes, below the limit of detection by RT-qPCR. We later confirmed these expression patterns by transcriptome deep sequencing (RNA-seq) (see below).

### Functional validation of *MG1*

We introduced targeted mutations in each candidate gene in ZH11 using clustered regularly interspaced short palindromic repeats (CRISPR)/CRISPR-associated protein 9 (Cas9)-mediated gene editing. We designed two sequence-specific single guide RNAs (sgRNAs) to disrupt their coding sequences (CDSs) (Fig. 2e, Supplementary Fig. 7a). We sequenced the target sites in all transgenic plants and used homozygous progeny carrying insertions/deletions (InDels) or a deletion between the two target sites for nematode inoculation (Fig. 2e, Supplementary Fig. 7c). Compared to wild-type ZH11, all independent *C61*-knockout plants were highly susceptible to *M. graminicola*, similar to Nipponbare, and bore significantly more galls (up to 9.8-fold) and nematodes than ZH11 (Fig. 2g, h). By contrast, CRISPR-edited plants for *C3*, *C4*, *C5*, *C9*, and *C62* retained the full resistance phenotype characteristic of ZH11 (Fig. 2h, Supplementary Fig. 7c, d). We concluded that *C61* is the *M. graminicola* resistance gene *MG1*.

We also sequenced the genomic region corresponding to *C61* from the resistant varieties SL 22-620 and HKG 98 and determined that the nucleotide and deduced amino acid sequences of *C61* are identical to ZH11, suggestive of the same origin for this locus. We then created *C61*-knockout lines in the SL 22-620 and HKG 98 backgrounds. Plants homozygous for the *C61*-knockouts in SL 22-620 and HKG 98 bore more galls (36.6- and 21.3-fold), compared to the corresponding wild-type (Fig. 2i, Supplementary Fig. 8a, b), further validating that *MG1* is required for *M. graminicola* resistance in ZH11, SL 22-620, and HKG 98.

### MG1 is a unique CC-NB-LRR protein

**Fig. 3:**
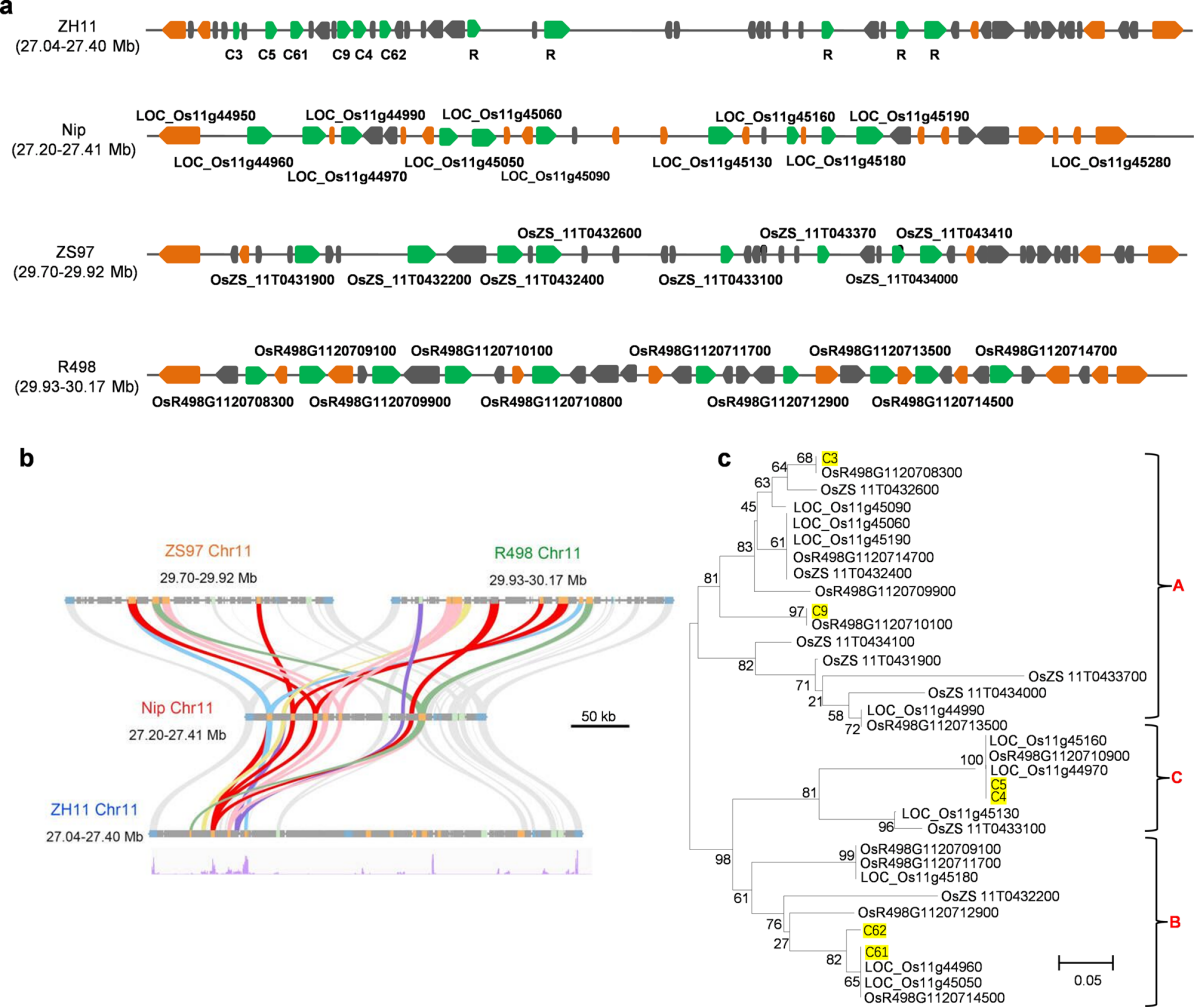
*MG1* encodes a CC-NB-LRR protein. **a** Gene arrangement of the *MG1* locus in ZH11, Nipponbare, ZS97, and R498 rice varieties. Green arrows indicate the predicted *NLR*s, and orange arrows represent expressed genes. The transposable elements are indicated in gray. **b** Colinearity analysis of *NLR* genes at the *MG1* locus between the four rice varieties. Gene expression levels in this region are derived from RNA-seq analysis and shown at the bottom. **c** Phylogenetic analysis of MG1 homologs in different rice varieties. The phylogenetic tree was generated using the neighbor-joining method. The bootstrap values are shown as a percentage next to the branches. Microscale unit is as indicated. The genes from ZH11 are colored in yellow.

*MG1* encodes a protein of 1,033 amino acids with a coiled-coil (CC) domain, an NB-ARC (APAF1, R gene products, and CED-4) domain, and an LRR domain (Supplementary Fig. 9a). The NB-ARC domain contained the conserved motifs P-loop, Resistance Nucleotide Binding Site (RNBS)-A, Kinase-2/Walker B, RNBS-B, GLPL, RNBS-D, and MHD (Supplementary Fig. 9b). *MG1* was located in a complex *NLR* gene cluster with large structural variation between rice genotypes. Therefore, we compared the chromosomal organization of *NLR* genes within this locus among the resistant variety ZH11, and three susceptible varieties Nipponbare, ZS97, and R498 with high-quality reference genomes (Fig. 3a, Supplementary Fig. 10). Ten *NLR*s were dispersed in Nipponbare (over a 210-kb interval) and R498 (240 kb), but eight and eleven in the same region of ZS97 (220 kb) and ZH11 (360 kb), respectively. Gene colinearity analysis illustrated the highly diversified *NLR* gene family between rice varieties (Fig. 3b), making it difficult to distinguish orthologous relationships among homologs within this locus. Phylogenetic analysis using amino acid sequences encoded by all *NLR* genes mentioned above identified three major groups of resistance (*R*) genes (A, B, and C), further demonstrating the high diversity among homologs (Fig. 3c).

### MG1 confers resistance to *M. graminicola*

**Fig. 4:**
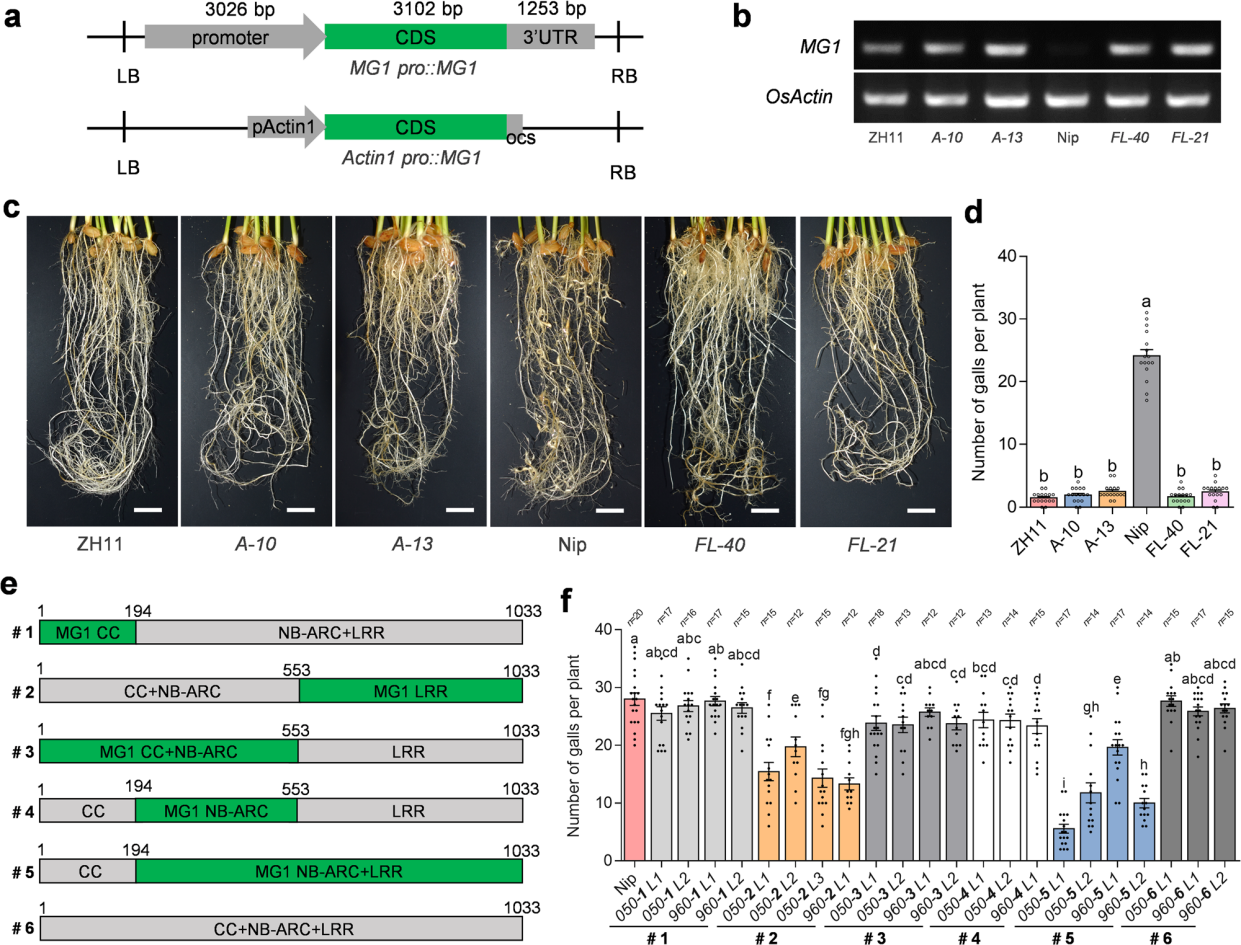
MG1 confers resistance to *M. graminicola*. **a** Schematic diagram showing the *MG1* complementation and overexpression cassettes used to transform Nipponbare. **b** RT-PCR analysis of *MG1* expression in the transgenic lines. *OsActin* was used as an internal control. *A-10* and *A-13* are overexpression lines. *FL-40* and *FL-21* are complementation lines. **c** Disease symptoms of transgenic lines at 15 dpi with *M. graminicola*. Scale bars, 2 cm. **d** Number of galls on the transgenic lines. Data are means ± s.e.m. (*n* = 15 independent plants) collected at 15 dpi. **e** Schematic diagram of domain-swap constructs between MG1 (green box) and LOC_Os11g44960 or LOC_Os11g45050 (gray box). #1–#6 on the left indicate different chimeric fragments. **f** Number of galls on transgenic plants transformed with individual chimeric construct as indicated in (**e**). The labels under the x-axis indicate LOC_Os11g44960 or LOC_Os11g45050 used for the domain-swap. Data are means ± s.e.m. collected at 15 dpi. Exact sample sizes (*n* = biologically independent plants) are given above the plot. Different letters above the bars in (**d**, **f**) indicate statistical significance groups at *P* < 0.05 (one-way ANOVA analysis followed by Fisher’s LSD multiple comparison test). Exact *P* values are provided in the Source Data file. The experiments (**c**, **d**, **f**) were performed three times with similar results.

To confirm the resistance function of MG1, we transformed a 7.4-kb genomic fragment of *C61* with its promoter into the susceptible variety Nipponbare (Fig. 4a). Homozygous T_2_ transgenic plants derived from independent T_0_ transformants were highly resistant to *M. graminicola*, similar to ZH11, compared to wild-type Nipponbare (Fig. 4b–d). We also overexpressed the *C61* coding sequence in Nipponbare under the control of the rice *Actin1* promoter (Fig. 4a, b). The resulting independent homozygous T_2_ transgenic lines displayed enhanced resistance to *M. graminicola* compared to the wild-type Nipponbare (Fig. 4c, d). Thus, we concluded that *C61* confers nematode resistance and is *MG1*.

To facilitate breeding of new resistant cultivars, we designed an InDel marker, CR24, and a cleaved amplified polymorphic sequences (CAPS) marker, WXM1, flanking or within the *MG1* locus, that co-segregates with *MG1*, as allele-specific molecular markers (Supplementary Fig. 11a). In addition to ZH11, SL 22-620, and HKG 98, two previously identified resistant varieties (LD24 and KPM) harbored an *MG1* resistance allele, while Toga and the other susceptible varieties did not (Supplementary Fig. 11b, c). Toga showed no correlation between its resistance phenotype and the *MG1*-associated genotypes, consistent with our previous finding that Toga contains a resistant locus distinct from *MG1*. To evaluate the potential utility of *MG1* in rice breeding, we introgressed the *MG1* locus into the susceptible variety Huazhan, which is commonly used as a restorer line in rice breeding programs, through successive backcrossing. The resulting near-isogenic line, NIL-*MG1*, was highly resistant to nematode infection, similar to ZH11 (Supplementary Fig. 11d, e). Moreover, *MG1* had no adverse effect on plant growth or yield traits (thousand-grain weight) under field conditions without infection in NIL-*MG1* (Supplementary Fig. 11f, g). The NIL-*MG1* displayed better growth in nematode-infested soil under greenhouse conditions, but whether the rice yield will be altered under infested field conditions needs to be determined in future (Supplementary Fig. 11h).

A phylogenetic analysis showed that MG1 clusters with LOC_Os11g44960 and LOC_Os11g45050 in Nipponbare, sharing 93.9% and 93.8% sequence identity, respectively (Fig. 3c, Supplementary Fig. 12). Amino acid alignment revealed 31 and 63 amino acid substitutions between MG1 and LOC_Os11g44960 or LOC_Os11g45050, respectively (Supplementary Fig. 12). When comparing MG1 with both susceptible alleles in Nipponbare, we identified SNPs causing 15 amino acid substitutions, all of which occurred within the LRR domains, especially the last two LRRs. To determine which of the MG1 domains are responsible for nematode resistance, we generated a series of chimeric constructs by replacing the sequence for different MG1 domains with the corresponding domains from LOC_Os11g44960 and LOC_Os11g45050 (indicated as #1-#6 in Fig. 4e) and expressed them in Nipponbare under the *MG1* promoter of ZH11 (Fig. 4e). We then evaluated nematode resistance in homozygous transgenic lines for each chimeric construct. All chimeric genes were expressed to similar levels (Supplementary Fig. 13). Neither LOC_Os11g44960 nor LOC_Os11g45050 (#6) conferred nematode resistance (Fig. 4f). Only two chimeric constructs (#2 and #5) encoding the LRR or NB-ARC-LRR domain of MG1, but none of the other domain combinations tested, enhanced nematode resistance, indicating that the LRR domain is critical for the activation of MG1 and nematode resistance.

Effector-triggered immunity mediated by R proteins is often accompanied by cell death. Isolated CC and NB domains, or the full length of some NLR proteins, activate cell death in the absence of the corresponding avirulence factors when their encoding genes are transiently expressed in *Nicotiana benthamiana*^29,30^. To examine whether MG1 similarly induces cell death, we individually expressed full-length *MG1* or the sequence encoding the CC, NB, and LRR domains in *Agrobacterium tumefaciens*–infiltrated *N. benthamiana* leaves. Although all proteins accumulated in the infiltrated leaves, none induced cell death (Supplementary Fig. 14a, b). Mutations in the MHD motif of the NB-ARC domain result in autoactivation of R proteins^31^. We therefore changed an aspartate to valine in the MHD motif of MG1 (MG1^D493V^) and expressed the corresponding construct in *N. benthamiana* leaves, which clearly induced HR-like cell death at 3 dpi (Supplementary Fig. 14a). Taken together, these results indicate that the activation of MG1 may be sufficient to trigger defense responses accompanied by cell death.

### Expression analysis of *MG1*

**Fig. 5:**
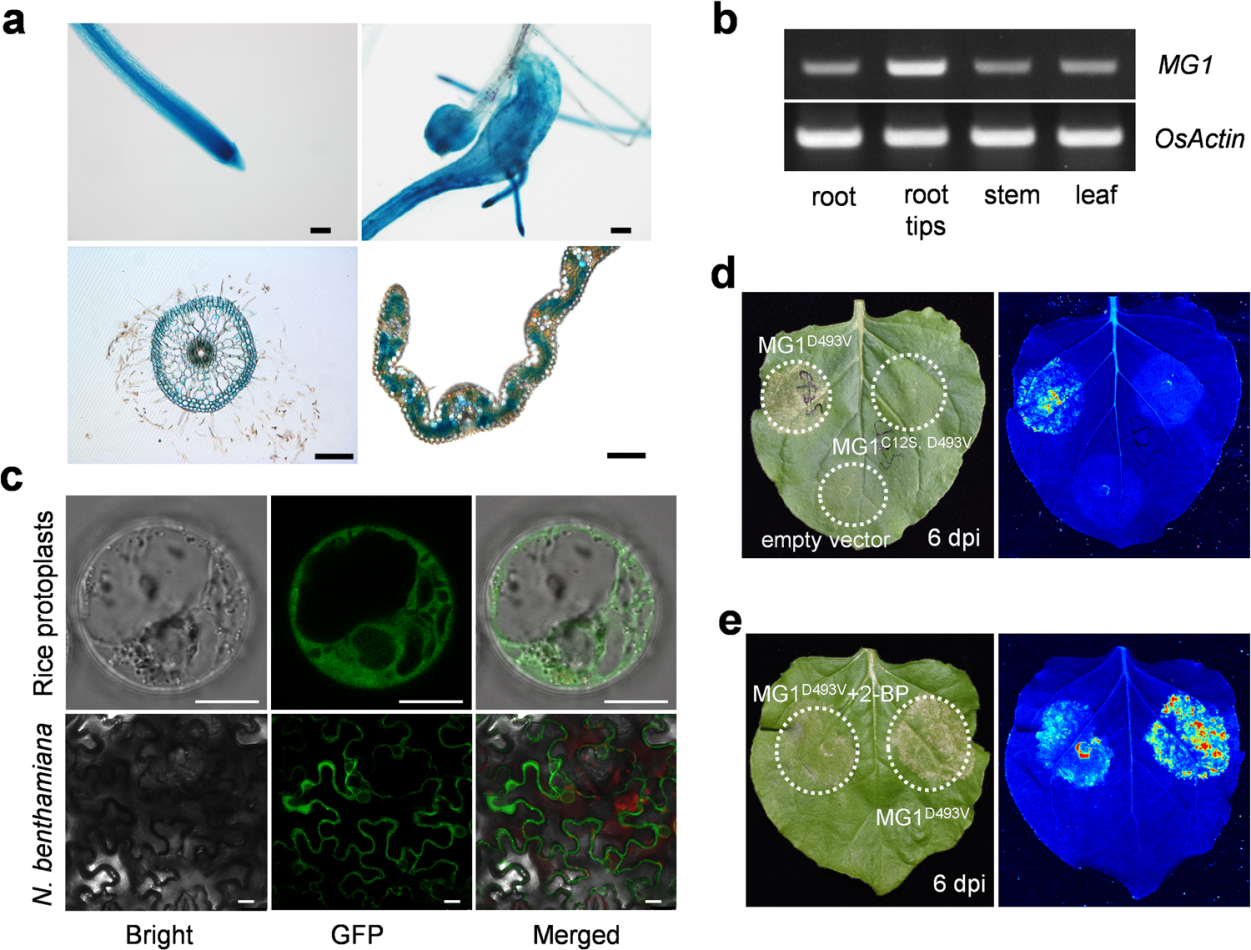
*MG1* expression and MG1 subcellular localization. **a** *MG1* promoter-*GUS* expression pattern. Upper panel shows root tips without (left) and with (right) nematode infection. Lower panel shows GUS activity in free-hand sections of roots and leaf blades. Scale bars, 100 μm. **b** RT-PCR analysis of *MG1* expression in different ZH11 tissues. *OsActin* was used as an internal control**. c** Subcellular localization of MG1. The *MG1-GFP* fusion construct was transiently transfected in rice protoplasts (upper panel) or infiltrated in *N. benthamiana* leaves (lower panel). Pictures were taken 16 h after protoplast transfection or 48 h post infiltration of *N. benthamiana*. Scale bars, 10 μm. **d** The cell death phenotype in *N. benthamiana* leaves infiltrated with *MG1*^*D493V*^ is abolished in *MG1*^*C12S, D493V*^. **e** Reduced cell death in *N. benthamiana* leaves infiltrated with *MG1*^*D493V*^ by treatment with the palmitoylation inhibitor 2-BP. Representative leaves were photographed under normal light (left) and UV light (right) at 6 days post infiltration (**d**, **e**). All experiments were performed three times with similar results.

Since typical hook-shaped galls form at the root tips of *M. graminicola*–infected rice plants, we investigated the *MG1* expression pattern in ZH11 with a *MG1* promoter-*β-glucuronidase* (*GUS*) reporter construct. We used an ~3-kb promoter fragment upstream of the *MG1* start codon to drive *GUS* expression and transformed the resulting construct into ZH11 to generate stable transgenic lines. We predominantly detected GUS staining in leaf mesophyll cells, root tips, and vascular tissues (Fig. 5a). Consistent with its role in recognizing nematode attack, we observed high GUS expression in galls upon *M. graminicola* infection. In addition, RT-PCR analysis revealed that *MG1* is constitutively expressed in roots, stems, and leaves, with the highest expression levels reached in root tips (Fig. 5b).

To determine MG1 subcellular localization, we transiently expressed a construct encoding an MG1-green fluorescent protein (GFP) fusion protein in rice protoplasts and *N. benthamiana*. We mainly observed fluorescence signals in the cytoplasm (Fig. 5c). The CC-NB-LRR protein ZAR1 (HOPZ-ACTIVATED RESISTANCE 1) forms a resistosome in the plasma membrane to trigger immunity and cell death^32,33^. Therefore, to further explore MG1 localization, we searched for potential modifications in MG1 that promote association with the plasma membrane. The online software CSS-Palm predicted the cysteine 12 residue in the MG1 N-terminal CC domain as a potential site for palmitoylation (Supplementary Table 3). To assess the importance of this residue in *MG1*-mediated nematode defense responses, we introduced a C12S mutation into the autoactivated D493V mutant of MG1 (MG1^C12S, D493V^) and tested its effect on cell death. The C12S mutation completely abolished the cell death–inducing capacity of MG1^D493V^ (Fig. 5d). Consistent with this result, treating *N. benthamiana* leaves infiltrated with the *MG1*^*D493V*^ construct with the palmitoylation inhibitor 2-bromopalmitate (2-BP) suppressed the cell death phenotype (Fig. 5e), while no obvious inhibitory effect on cell death caused by another R gene *RLS1*^34^ (Supplementary Fig. 15). These results indicated that the C12 residue in the MG1 N-terminal CC domain, a potential palmitoylation site, is required for the cell death activity of MG1 (Fig. 5e).

### Rice resistance responses to *M. graminicola* infection

**Fig. 6:**
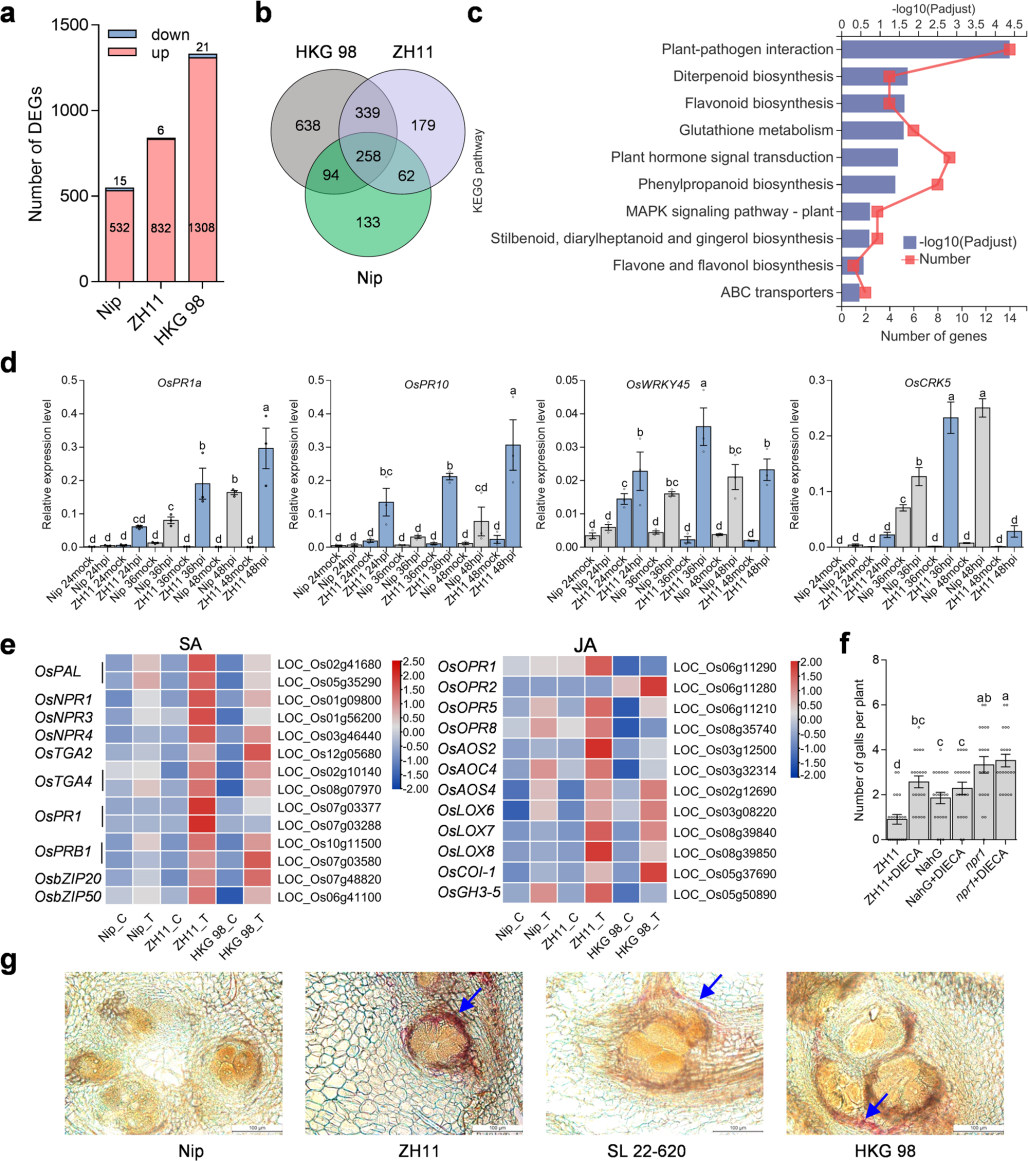
Resistance responses of *MG1*-carrying rice varieties upon *M. graminicola* infection. **a** Number of DEGs in susceptible (Nipponbare) and resistant (ZH11 and HKG 98) rice varieties at 1 day post nematode infection. **b** Venn diagram displaying the common DEGs between Nipponbare, ZH11, and HKG 98. **c** KEGG enrichment analysis of common DEGs upregulated in resistant varieties. **d** Expression analysis of defense-related genes in Nipponbare and ZH11. Nematode-infected roots were collected for RT-qPCR at 24, 36, and 48 hpi. Data are means ± s.e.m. of three independent biological replicates. **e** Heatmap representation of the expression levels of SA and JA pathway–related genes in ZH11 and Nipponbare. Nip_C, ZH11_C, and HKG 98_C represent control samples. Nip_T, ZH11_T, and HKG 98_T represent nematode-treated samples. The TPM values were normalized in the row direction. Red or blue color indicates relatively high or low expression, respectively. **f** Number of galls on *NahG* and *npr1* plants with or without treatment of the JA biosynthesis inhibitor DIECA. Two-week-old plants were treated with 100 μM DIECA 24 h before nematode inoculation. Data are means ± s.e.m. from one representative experiment (*n* = 21 independent plants). **g** Lignin staining of nematode-induced root galls in different rice varieties. Sections were stained with Wiesner reagent at 5 dpi. Scale bar, 100 μm. Different letters above the bars in (**d**, **f**) indicate statistical significance groups at *P* < 0.05 (one-way ANOVA analysis followed by Fisher’s LSD multiple comparison test). Exact *P* values are provided in the Source Data file. The experiments were performed three times with similar results (**f**, **g**).

To understand the underlying resistance mechanism mediated by MG1, we performed a global comparative transcriptome profiling between the resistant (ZH11 and HKG 98) and susceptible (Nipponbare) varieties at the early stage (1 dpi) of nematode infection. Analysis of all differentially expressed genes (DEGs) suggested that the resistant varieties display an overall enhanced nematode response compared to Nipponbare (Fig. 6a, Supplementary Fig. 16a). We investigated the 339 genes co-upregulated in the resistant varieties using Gene Ontology (GO) enrichment analysis and observed a significant overrepresentation (*P*-adjust <0.05) for genes involved in defense response, protein phosphorylation, kinase activity, and response to stress (Fig. 6b, Supplementary Fig. 16b). Based on a similar pathway enrichment analysis via Kyoto Encyclopedia of Genes and Genomes (KEGG), we determined that plant–pathogen interaction, biosynthesis of secondary metabolites, plant hormone signal transduction, and MAPK signaling pathway are enriched in the resistant varieties (Fig. 6c). We also investigated the expression patterns of the defense-related genes *PATHOGENESIS-RELATED GENE 1a* (*OsPR1a*), *OsPR10*, *CDPK-RELATED PROTEIN KINASE 5* (*OsCRK5*), and *OsWRKY45* in ZH11 and Nipponbare at different time points following nematode inoculation by RT-qPCR. We detected a more rapid and much stronger induction of gene expression in ZH11 compared to Nipponbare (Fig. 6d).

The importance of jasmonate (JA) and salicylic acid (SA) hormone pathways for nematode resistance is well established in compatible plant–nematode interactions^35,36^. We thus explored the roles of JA and SA in MG1-mediated nematode resistance. Genes involved in SA and JA biosynthesis and signaling were differentially activated upon nematode infection, with much stronger induction was observed in resistant genotypes (Fig. 6e). Notably, the SA-deficient transgenic *NahG* line and the SA-signaling mutant *npr1* in the ZH11 background had more galls and lower resistance against *M. graminicola*. Chemical inhibition of JA biosynthesis by exogenously applying diethyldithiocarbamic acid (DIECA) also compromised the nematode resistance of wild-type ZH11, but we observed no additive effect for the SA-defective mutants (Fig. 6f). These data indicate that SA and JA are required for MG1-mediated resistance.

Lignin and callose accumulate as physical barriers following nematode infection^37^. To investigate the defense responses in the resistant and susceptible lines, we assessed lignin and callose staining in nematode-infected root galls at 3 dpi. We observed stronger red staining surrounding the feeding cells in the resistant varieties compared to Nipponbare, suggesting increased lignin accumulation in the resistant varieties (Fig. 6g). Callose deposition, seen as many bright speckles, also increased in the resistant varieties (Supplementary Fig. 16c). In agreement, RNA-seq revealed that genes related to lignin and callose biosynthesis are differentially regulated by nematode invasion between resistant and susceptible genotypes (Supplementary Fig. 16d). Taken together, global gene expression profiles and histological analyses indicated that ZH11 and HKG 98 undergo a series of resistance responses to prevent nematode parasitism at the early stage of infection.

### MGBP1 contributes to MG1-mediated nematode resistance

**Fig. 7:**
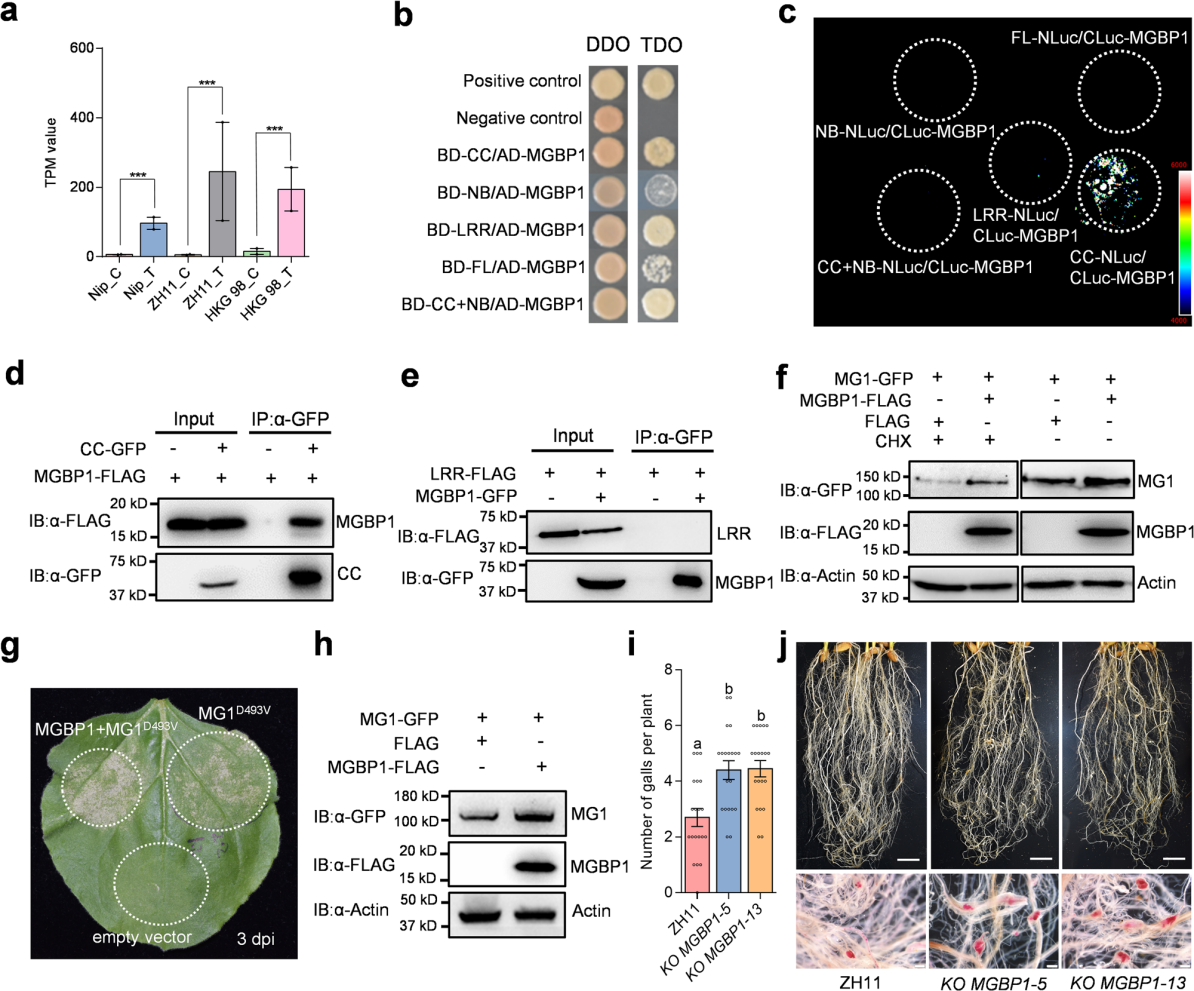
MGBP1 directly interacts with MG1 in MG1-mediated nematode resistance. **a** Expression levels of *MGBP1* retrieved from RNA-seq data. Asterisks indicate significant differences between the control (C) and the treated (T) samples (***P < 0.001). **b** The MG1 CC and LRR domains interact with MGBP1 in the Y2H assays. Combinations of AD/SV40 with BD/p53 and BD/Lam served as positive and negative controls, respectively. The interaction was tested on synthetic defined (SD) medium lacking His, Leu, and Trp (SD–H–L–T). **c** The MG1 CC domain interacts with MGBP1 *in planta*. Split-luciferase assays were conducted with the indicated constructs in *N. benthamiana*. **d**, **e** The CC domain, but not the LRR domain, of MG1 interacts with MGBP1 by Co-IP assay. The indicated constructs were infiltrated in *N. benthamiana*. Anti-GFP-conjugated beads were used for immunoprecipitation (IP: α-GFP), and anti-GFP or anti-FLAG antibody was used for detection (IB: α-GFP or IB: α-FLAG). **f** The effect of MGBP1 on MG1 abundance in *N. benthamiana*. *MG1-GFP* and *MGBP1-FLAG* or the empty vector control were co-infiltrated in *N. benthamiana* with or without 50 μM CHX 4 h before immunoblot analysis. The proteins were detected with anti-GFP and anti-FLAG antibodies, respectively. Equal loading was confirmed by an anti-Actin immunoblot. **g** Enhanced cell death of *N. benthamiana* leaves co-infiltrated with *MG1*^*D493V*^ and *MGBP1*. **h** The effect of MGBP1 on MG1 abundance in rice protoplast. *MGBP1-FLAG* or the empty vector control was transfected into the protoplast derived from *MG1-GFP* transgenic plants. The proteins were detected with anti-GFP and anti-FLAG antibodies, respectively. **i** Number of galls on *MGBP1*-knockout mutant plants at 15 dpi. Data are means ± s.e.m. from one representative experiment (*n* = 20 independent plants). Different letters above the bars indicate statistical significance groups at *P* < 0.05 (one-way ANOVA analysis followed by Fisher’s LSD multiple comparison test). **j** Representative disease symptoms of *MGBP1*-knockout mutant roots after infection with *M. graminicola*. Roots were stained with acid fuchsin at 15 dpi. Scale bars, 2 cm (upper panel), 500 μm (lower panel). The experiments were performed three times with similar results (**b**–**j**). Exact *P* values are provided in the Source Data file (**a**, **i**).

To dissect the signaling components involved in MG1-mediated resistance, we performed a yeast two-hybrid (Y2H) screen using the MG1 CC or LRR domains as bait. Among ten candidates we obtained after initial screening, we focused on MG1-BINDING PROTEIN 1 (MGBP1, LOC_Os12g25090) for further characterization (Supplementary Table 4). MGBP1 is a small protein of 100 amino acids in the potato type I serine protease inhibitor family. No sequence variation is found for MGBP1 in resistant and susceptible varieties. *MGBP1* expression was significantly upregulated upon nematode infection, with higher expression seen in the resistant varieties (Fig. 7a). Yeast colonies co-transformed with constructs encoding MGBP1 and the LRR, CC-NB, or CC domain grew well on selective medium (Fig. 7b). A luciferase complementation imaging (LCI) assay in *N. benthamiana* validated the interaction between MGBP1 and the MG1 CC domain, but not with the other MG1 domains tested (Fig. 7c). A co-immunoprecipitation (Co-IP) assay also supported this specific interaction. Following immunoprecipitation with an anti-GFP antibody, we detected MGBP1-FLAG with an anti-FLAG antibody in protein extracts from *N. benthamiana* leaves co-infiltrated with *CC-GFP* and *MGBP1-FLAG* constructs but not in the control (Fig. 7d). We detected no interaction between MGBP1 and the MG1 LRR domain in this assay (Fig. 7e).

Since protease inhibitors are involved in protein turnover, we tested the influence of MGBP1 on MG1 accumulation. Co-expressing *MG1* and *MGBP1* constructs in *N. benthamiana* leaves raised MG1 abundance (Fig. 7f). In line with this observation, MG1^D493V^-triggered cell death was stronger when *MG1*^*D493V*^ was co-expressed with *MGBP1* relative to *MG1*^*D493V*^ alone (Fig. 7g). Consistently, we observed the increased protein level of MG1 when *MGBP1* is co-expressed in rice protoplasts (Fig. 7h). To explore whether MG1-mediated resistance depends on MGBP1, we used CRISPR/Cas9-mediated editing to mutate *MGBP1* in the ZH11 background (Supplementary Fig. 17) and obtained two independent T_2_ homozygous mutants for nematode resistance. Compared to ZH11, the edited *mgbp1* mutants had significantly reduced resistance to *M. graminicola*, although they were not as susceptible as Nipponbare (Fig. 7i, j). These results suggest that MGBP1 contributes to MG1-mediated defense responses, probably by affecting protein levels of MG1 or serving as a potential guardee of nematode effectors to achieve effective resistance.

## Discussion

With climate change, water shortages, and changes in rice cultivation systems, the incidence of *M. graminicola* is increasing, especially in Asia. Mining resistance genes for crop disease improvement is an environmentally friendly and practical solution to mitigate this concern. Here, we evaluated the response of 207 rice varieties to this nematode under controlled conditions, and four varieties (Toga, SL 22-620, HKG 98, and ZH11) displayed high resistance. *MG1*, a dominant resistance gene on chromosome 11, is the common resistance gene in SL 22-620, HKG 98, and ZH11. *MG1* introgressed into the susceptible rice cultivar Huazhan significantly enhanced its resistance to *M. graminicola*, comparable to resistant varieties. Thus, *MG1* and the *MG1*-linked molecular markers developed here will have great value in marker-assisted breeding. Furthermore, Toga, which carries a distinct resistance gene and does not carry the resistant allele of the *MG1* locus, provides another resource for breeding rice with durable nematode resistance through resistance-gene pyramiding or diversification.

*NLR* genes are enriched on rice chromosome 11 (Rice Chromosome 11 and 12 Sequencing Consortia 2005), and many of them are clustered. We located *MG1* in a complex *NLR* gene cluster on chromosome 11 and observed large structural variations between rice varieties. This locus, previously referred to as AC134922, is one of the most diversified *R* gene loci in rice genomes^38^. *R* genes undergo adaptive evolution in response to pathogens, and their clustering may increase the opportunity for genetic exchange, acting as potential resistance reservoirs against future pathogen strains^39^. Retrotransposons, which are abundant at this locus, may be an important driving force in *R* gene evolution and diversication^40^. Comprehensive sequence comparison across diverse rice varieties will help to better understand the evolutionary mechanisms acting on *R* genes. Whether other *MG1* homologs are responsible for disease resistance remains to be studied. We established, through bioinformatic sequence analysis and marker-assisted screening, that few rice varieties belonging to *japonica*, *indica*, and *aus* subspecies carry *MG1*, suggesting that *MG1* has not been widely selected for during rice breeding. We propose that the *MG1* locus from the resistant varieties shares the same origin, but how this specific *MG1* locus was introgressed into rice varieties distributed in different regions of the world is still elusive.

Plants rely on membrane-associated pattern recognition receptors (PRRs) and intracellular NLRs to activate the two tiers of plant immune responses, pattern-triggered immunity (PTI) and effector-triggered immunity (ETI), to defend against various pathogens^41,42^. By directly or indirectly recognizing pathogen effector proteins, NLRs initiate robust disease resistance responses that typically result in localized host cell death^43,44^. The LRR domain of NLRs mainly mediates direct or indirect pathogen effector sensing^45,46^. *MG1* encodes a CC-NB-LRR-type protein, and the expression of its susceptible alleles failed to trigger nematode resistance. Domain swap analysis between MG1 and its susceptible orthologs demonstrated the pivotal role of the MG1 C-terminal LRR domain in conferring nematode resistance. The MG1 LRR domain contains many amino acid substitutions when compared to its susceptible alleles in Nipponbare, indicating that potential adaptive divergence in the LRR domain may alter pathogen recognition specificity. Taken together, we hypothesize that the MG1 LRR domain determines the specificity of nematode recognition and is largely implicated in MG1 activation.

Plant parasitic nematodes deliver effectors into the host cells through their stylet to support parasitism. Nematodes are attracted to and penetrate roots of resistant plants; however, the development of the feeding site is substantially hindered, and the nematodes starve to death or leave the roots^47,48^. Resistant plants initiate a localized HR near the feeding site in roots. However, how resistance genes are activated is not well understood. The root-knot nematode effectors Map-1 and Cg-1 may be involved in resistance mediated by the resistance gene *Mi-1*, but further validation is needed^49,50^. Direct evidence of nematode effectors implicated in ETI comes from potato cyst nematode (*Globodera rostochiensis* and *Globodera pallida*)-secreted effectors RAN-BINDING PROTEIN LIKE-1 (GpRBP1) and VENOM-ALLERGEN PROTEIN 1 (GrVAP1), which are directly or indirectly recognized by the R proteins Gpa2 and Cf-2, respectively^51,52^. To develop rice varieties with durable nematode resistance, it will be crucial to identify the corresponding avirulence effectors of MG1 and to elucidate the MG1 mechanisms for perceiving nematode presence.

In the absence of pathogens, NLR proteins are repressed through self-inhibitory intramolecular interactions. It is crucial for plants to tightly control the NLR protein state to avoid fitness penalties. The presence of effectors induces a conformational change, which releases the intramolecular interactions to activate NLR signaling^53,54^. We provided evidence that MG1-mediated resistance is highly dependent on the presence of nematode. First, we detected high *MG1* expression at the nematode invasion site, although this was not influenced by nematode invasion, suggesting that MG1 may be inactive prior to nematode infection. The perception of the cognate effectors secreted from nematodes may therefore be a prerequisite for MG1 activation. Second, neither MG1 nor its individual domains triggered cell death in *N. benthamiana* leaves, in contrast to the cell death phenotype observed for the autoactivated form of MG1 (MG1^D493V^) and NLRs like Brown Planthopper Resistance Gene 9 (BPH9), RESISTANT TO P. SYRINGAE 5 (RPS5), MILDEW A (MLA), Stem rust 33 (Sr33), and Sr50^30,55^. Furthermore, the MG1 CC domain self-associated, similar to other NLRs (Supplementary Fig. 18a, b), whose self-association or oligomerization is key for NLR activation^32,56^. Recent breakthroughs on the CNL (CC-containing NLR) protein ZAR1 demonstrated that rearranging the CC domain leads to oligomeric resistosomes at the plasma membrane, serving as calcium-permeable cation-selective channels that trigger immune signaling and cell death^32,33,53^. In addition, a cysteine at position 12 of MG1, which was predicted as a palmitoylation site, was required for cell death activity. Palmitoylation-dependent membrane localization of Pit, a rice blast resistance protein, may activate rice immunity^57^. Our data suggest that palmitoylation-mediated membrane localization might be also required for MG1 function. Finally, our results indicate that similar defense responses are activated in resistant plants during incompatible interactions with pathogens, nematodes, and insects^58^. Indeed, SA and JA pathways were required for MG1-mediated resistance, and lignin and callose deposition was highly induced at the nematode feeding site in resistant rice varieties. These transcriptome and cytological changes likely facilitate a robust response to nematode threats. Our results corroborate previous reports that phytohormone pathways and secondary metabolites contribute to nematode resistance in compatible rice–nematode interactions^35^.

*NLR*s are a class of resistance genes widely used in crop disease resistance breeding. Our current understanding of NLR signaling mechanisms in crops is very limited. We revealed that MGBP1, a member of the potato type I serine protease inhibitor family, contributes to MG1-mediated nematode defense responses and can directly interact with MG1. *MGBP1* was highly expressed in resistant plants compared to susceptible Nipponbare upon nematode infection. Protease inhibitors inactivate proteases to regulate protein degradation or turnover and combat insect and nematode infection by inhibiting their digestive enzymes^59^. Enhanced resistance to several nematode species by expressing protease inhibitor genes has been described in various host plants^60,61^. Whether MGBP1 has nematocidal activity deserves further investigation. Protease inhibitors are also involved in endogenous physiological processes, such as plant growth and development, seed germination, senescence, and programmed cell death, by counteracting the activities of specific proteases^62,63^. Here, we observed the higher accumulation of MG1 when co-expressed with a construct encoding MGBP1, although we cannot rule out the possibility that MGBP1 might similarly affect the levels of other defense components in response to nematode infection. Further characterization using a proteomics approach might help address this question. In addition, whether nematode-secreted peptidases target MG1 to evade immunity is unknown. It’s plausible that MG1 guards MGBP1 from potential perturbations by nematode effectors during infection. Thus, the underlying molecular mechanism of how MGBP1 contributes to the defense response remains to be elucidated.

## Methods

### Plant and nematode materials

The rice (*Oryza sativa* L.) lines used in this experiment are listed in Supplementary Table 1 and include 197 varieties from the United States Department of Agriculture (USDA) Rice Mini-Core Collection and 10 rice varieties maintained in our laboratory. All rice materials were originally provided by Dr. Chengcai Chu at the Institute of Genetics and Developmental Biology (IGDB), Chinese Academy of Sciences (CAS). The SA-deficient NahG transgenic line and the *npr1* mutant in the ZH11 background were kindly provided by Dr. Jiuyou Tang (IGDB, CAS). The *Meloidogyne graminicola* culture was provided by Dr. Gaofeng Wang at Huazhong Agricultural University (HZAU) and was originally isolated from a rice field from Haikou in Hainan Province, China. A pure nematode culture was maintained on Nipponbare (*O. sativa*) growing in a sand:potting soil mixture (3:1) at 26–28 °C under a 16-h-light/8-h-dark regime with 250 μmol m^–2^ s^–1^ light intensity and 70–75% relative humidity. Four weeks after inoculation, infected roots were cut into 1-cm pieces and ground with a blender. The slurry was washed through a stack of sieves (mesh number: #60, #200, and #500). Eggs collected from the bottom sieve were incubated in a hatching chamber with distilled water at 28 °C for 1 day. Freshly hatched second-stage juveniles (J2s) were used for the experiments.

### Nematode infection experiments

Rice seeds were germinated at 28 °C for 2–3 days and then transferred to a sand:potting soil mixture for growth. The seedlings were watered daily and fertilized twice a week with Hoagland solution. Fourteen-day-old rice plants were inoculated with 150 *M. graminicola* J2s per plant or mock-inoculated with 0.1% (w/v) agarose. The infection level of the plants was evaluated at 15 dpi by counting the number of galls per plant. For the large-scale screening, 12 plants in two separate pots (six plants per pot) were assessed for each variety. Acid fuchsin staining was performed to visualize the galls^35^. Roots were boiled in 0.8% (v/v) acetic acid and 0.013% (w/v) acid fuchsin for 3 min and then destained in acidified glycerol. Nematode development was assessed under a stereomicroscope by counting the number of nematodes of various stages at the times indicated in the text.

### Nematode attraction assay

The nematode attraction assay was performed as previously described^64^. In brief, 1 mL of 23% (w/v) pluronic F-127 (Sigma-Aldrich) containing 1000 J2s was aliquoted into 12-well tissue culture plates at 4 °C. Then, a 1-cm-long root tip was placed into each well. The number of nematodes touching the terminal 1.5 mm of the root tip was scored at the indicated time points (2, 4, and 6 hpi). The experiment was performed three times with at least 12 roots for each treatment.

### RNA extraction and RT-qPCR analysis

Total RNA was isolated from rice roots using TRIpure reagent (Aidlab, Beijing, China), and the RNA concentration was quantified with a NanoDrop-1000 spectrophotometer (Thermo Scientific, USA). First-strand cDNA was synthesized using HiScriptII QRT SuperMix (Vazyme, Nanjing, China) following the manufacturer’s instructions. Gene-specific primers were designed and quantitative PCR was performed using AceQ qPCR SYBR Green Master Mix (Vazyme, Nanjing, China) with an iCycler iQ5 thermal cycler (Bio-Rad, California, USA) real-time PCR system. *OsEXPNAR* (LOC_Os07g02340) and *OsActin* (LOC_Os03g50885) were used as internal controls; the expression of reference genes was not influenced by various stress conditions, including nematode infection^65^. A dissociation curve was generated to verify amplicon specificity. The experiment was performed three times, and relative gene expression was determined using the 2^–ΔCt^ method compared to the internal control^66^. The primers used for qPCR are listed in Supplementary Table 5.

### RNA-seq and data analysis

Three rice varieties, Nipponbare, HKG 98, and ZH11, were used for RNA-seq analysis. Six-day-old rice seedlings were inoculated individually with 150 *M. graminicola* J2s suspended with 0.1% (w/v) agarose solution. Controls were mock-inoculated with agarose solution alone. At 1 dpi, root tips (2 mm in length) or visible galls were collected and frozen in liquid nitrogen for further use. Forty root samples were pooled for each treatment, and two biological replicates were performed. Total RNA was extracted using the Ultrapure RNA Kit (CWBIO, Beijing, China). RNA integrity was assessed using an RNA Nano 6000 assay kit on a Bioanalyzer 2100 system (Agilent Technologies, CA, USA). Library construction for mRNA sequencing was performed using the NEBNext Ultra RNA Library Prep Kit for Illumina (NEB, USA) following the manufacturer’s recommendations. The libraries were sequenced using an Illumina Hiseq platform as 100-bp single-end reads (Majorbio, Shanghai, China). The data are accessible through the National Center for Biotechnology Information (NCBI) accession number PRJNA907725. Raw reads were filtered by removing adapter sequences, reads containing poly-N, and low-quality reads (quality score <20). The resulting clean reads were aligned and assembled to the Nipponbare reference genome (MSU7 release from the http://rice.uga.edu) using TopHat v2.0.12 and Cufflinks. Then, transcripts per million reads (TPM) values for each gene were calculated based on the length of the gene and read counts mapped to this gene using RSEM software. Differential gene expression was determined using the DESeq2 R package (1.18.0) with Log_2_FC ≥ 1 and a false discovery rate (FDR) < 0.05 as the threshold. Values of Log_10_(TPM + 1) were used for hierarchical clustering analysis. The expression heatmap and KEGG enrichment analyses were generated with values of Log_2_FC using TBtools v1.0^67^. GO enrichment was determined using Goatools. The cutoff for significant enrichment was *P*-adjust <0.05.

### Mapping and cloning *MG1*

To map *MG1*, the mapping F_2_ populations derived from the crosses ZH11 × Lehui 188, ZH11 × MH63, and HKG 98 × Nipponbare were generated. Moreover, four F_2_ populations derived from the crosses SL 22-620 × ZH11, HKG 98 × ZH11, ZH11 × Toga, and HKG 98 × Toga were generated for allelism tests. Nematode resistance was scored by counting the number of galls per F_2_ individual. To confirm the phenotypes of the F_2_ individuals, their F_2:3_ progeny were evaluated. The CTAB method was used to extract DNA from fresh leaves of individual rice seedlings.

*MG1* was initially localized to a region on chromosome 11 by BSA. Briefly, resistant (R) and susceptible (S) DNA samples, extracted from 30 F_2_ individuals each with extremely resistant or susceptible phenotypes, were pooled and used as R and S bulk samples. A total of 134 simple sequence repeat (SSR) markers distributed evenly on all 12 rice chromosomes were used to screen polymorphisms between R and S pools as well as both parents. QTL-seq was conducted to detect resistance loci. Each of the DNA samples pooled above was used for whole-genome resequencing with >30× genome coverage. The data are available in NCBI under accession number PRJNA907931. The resulting short reads were aligned to the Nipponbare reference sequence using BWA software. The GATK toolkit was used for SNP calling. The SNP-index and derived ∆(SNP-index) were calculated according to the pipeline described by Takagi et al.^68^. Smoothed G′ value analysis was conducted according to Magwene et al.^29^. A QTL was identified as a peak or valley of the ∆(SNP-index) plot or G′ value plot. The x-axis corresponds to the chromosomal position. The ∆(SNP-index) plot (the 2nd top) is shown with statistical confidence intervals under the null hypothesis of no QTL (blue, *P* < 0.1; green, *P* < 0.05; red, *P* < 0.01). For G′ value and its corresponding *P*-value (–Log_10_ value) plot (bottom two), the red threshold line represents *q* < 0.01.

To fine-map *MG1*, more DNA markers primarily based on InDel polymorphisms and derived cleaved amplified polymorphic sequences (dCAPSs) were designed according to the published sequences of the *japonica* Nipponbare and ZH11 (http://www.mbkbase.org/) and the *indica* R498 and Minghui 63 (MH63) varieties (https://rice.hzau.edu.cn/rice_rs2/). We used the flanking markers Tj146-3 and Tj146-7 to screen ZH11 × Lehui 188 F_2_ plants, and the 214 obtained recombinants delineated *MG1* to a 1.37-Mb interval between markers WXM35 and 11-27 M. Similarly, *MG1* was delineated to a 640-kb interval between markers WXM37 and CR16 using 5792 F_2_ plants generated from the cross between HKG 98 and Nipponbare. The F_2_ and F_2:3_ populations consisting of 10,836 and 2137 individuals for the crosses of ZH11 × Lehui 188 (or MH63) and HKG 98 × Nipponbare were used for linkage analysis and identification of recombinants.

To identify all candidate genes, the coding regions (http://hollywood.mit.edu/GENSCAN.html) and protein structure (http://smart.embl-heidelberg.de/) of the target region were predicted. To determine the expression levels of these candidates, reads obtained from the RNA-seq were aligned and mapped to the target locus of ZH11 using TopHat and Cufflinks.

The near-isogenic lines carrying *MG1* (NIL*-MG1*) were generated by repeated backcrossing of the resistant parent ZH11 to the recurrent parent Huazhan and were selected from the BC_3_F_4_ population through marker-assisted selection. The tightly linked *MG1* markers and additional SSR markers were used to screen the resistance locus and genetic background. Huazhan and NIL-*MG1* plants were examined for nematode resistance and agronomic traits. The agronomic performance experiment was conducted in a standard paddy field of Wuhan, China. The plants were grown in a randomized block design with three replicates. At harvest, thousand-grain weight was measured.

All PCR primers and markers are listed in Supplementary Table 5.

### Sequence analysis

The J. Craig Venter Institute (JCVI) toolkit was used for genome assembly, annotation, and comparative genomics^69^. We used the default parameters of this toolkit to draw a colinearity plot to compare the distribution of the *MG1*-related *NLR* gene cluster on chromosome 11 between four rice varieties (ZH11, Nipponbare, ZS97, and R498). The coding sequences of the *NLR* genes within the cluster from ZH11 were used to identify the colinear orthologs. Protein sequences within the *NLR* gene cluster were obtained for different rice varieties. ClustalX v2.1 was used to align the amino acid sequences. Conserved domains were predicted by BlastP, SMART (http://smart.embl-heidelberg.de/), and LRR search (https://lrrsearch.com/) online tools. The phylogenetic tree was generated with the predicted full-length amino acid sequences of NLR proteins using the MUSCLE algorithm of the phylogenetic analysis software MEGA7^70^. A bootstrap consensus tree was constructed inferred from 1000 replicates using the neighbor-joining method, and the *P*-distance model with complete gap deletion was used to calculate the evolutionary distances.

### Vector construction and transformation

To identify the full-length sequences of candidate genes, 5′ RACE and 3′ RACE were conducted. Total RNA was isolated from *M. graminicola*–infected ZH11 roots using the RNAprep pure Plant Kit (TIANGEN, China). 5′ RACE cDNA and 3′ RACE cDNA were synthesized according to Scotto-Lavino et al.^71^. Two rounds of amplification were performed using gene-specific primers and adaptor primers listed in Supplementary Table 5. The largest PCR products were cloned, and three individual clones were sequenced.

Targeted mutagenesis of candidate genes was performed using CRISPR/Cas9^72^. Briefly, two specific sgRNAs were designed for each gene. The polycistronic tRNA-gRNA (PTG) fragments were assembled using the Golden Gate assembly method and subsequently inserted into the pRGEB32 binary vector (courtesy of Kabin Xie at HZAU). The resulting constructs were introduced into rice variety ZH11 via *Agrobacterium tumefaciens*–mediated transformation according to a conventional protocol. The construct targeting *MG1* was also transformed into rice varieties HKG 98 and SL 22-620. Plants regenerated from hygromycin-resistant calli and their progeny were genotyped by PCR using primers flanking the target sites, and mutations were confirmed by sequencing. T_1_ or T_2_ homozygous transgenic lines derived from each independent T_0_ plant were used for the resistance test.

To construct the *MG1* complementation vector, a 7381-bp genomic fragment containing 3 kb of the promoter region, the entire coding region, and 3 kb of 3′-flanking sequence of *MG1* was amplified from ZH11 using gene-specific primers, and the PCR product was inserted into the binary vector pCAMBIA2300 (courtesy of Chengcai Chu at CAS) digested by *Sal*I and *Xba*I. The overexpression vector was constructed by sub-cloning the *MG1* coding sequence into the binary vector pCAMBIA2301 under the control of the rice *Actin1* promoter with *Sal*I and *Pst*I digestion. The constructs were introduced into susceptible variety Nipponbare via *A. tumefaciens*–mediated transformation to generate independent lines for functional verification of *MG1*. RT-qPCR was performed to evaluate gene expression levels.

For the domain swapping experiment, the sequences encoding various domains (CC, CC-NB-ARC, NB-ARC, NB-ARC-LRR, LRR, and the full-length protein) of MG1 or its two orthologs from Nipponbare (LOC_Os11g44960 and LOC_Os11g45050) were amplified. A series of chimeric fragments recombined at different positions between MG1 and both orthologs were generated by overlapping PCR. The resulting products and the *MG1* promoter were ligated into the pCAMBIA2300 binary vector with *Xba*I and *Sal*I restriction sites. The constructs encoding the MG1^D493V^ and MG1^C12S, D493V^ variants were generated using overlap extension PCR primers carrying point mutations, and the amplified products were cloned into the pCAMBIA1300-35S vector with *Xba*I and *Sal*I sites and verified by sequencing. These constructs were tested for cell death activity after transient infiltration in *Nicotiana benthamiana* leaves or transformed into Nipponbare for resistance function analysis.

A 3-kb promoter fragment upstream of the ATG start codon of *MG1* was amplified from ZH11 genomic DNA and cloned into the pCAMBIA2391Z vector with *Sal*I and *Eco*RI to build the *GUS* reporter construct. This *MG1pro:GUS* vector was introduced into ZH11, and T_2_ transgenic lines were used for subsequent analysis.

For subcellular localization, the *MG1* coding sequence was digested with *Xba*I and *Sal*I and inserted into the pCAMBIA2300-35S-GFP vector in-frame and upstream of *GFP*.

To generate constructs for Y2H assays, various fragments of *MG1* were cloned into the pGADT7 (AD) and pGBKT7 (BD) vectors. The full-length coding sequence of *MGBP1* was cloned in-frame and downstream of the sequence encoding the GAL4 DNA activation domain in the pGADT7 vector with *Nde*I and *Xho*I digestion.

For LCI assays, the full-length coding sequence of *MGBP1* was cloned into the pCAMBIA-35S-CLuc vector to form the CLuc-MGBP1 fusion construct. Different fragments of *MG1* were amplified and inserted into the pCAMBIA-35S-NLuc and pCAMBIA-35S-CLuc vectors to express C-terminal *NLuc* and N-terminal *CLuc* fusion constructs, respectively.

For Co-IP assays, the coding sequences being tested were separately cloned into pCAMBIA1300-35S-3×Flag and pCAMBIA2300-35S-GFP vectors using *Xba*I and *Sal*I sites to produce C-terminal-tagged fusion constructs.

All primers used to construct vectors are listed in Supplementary Table 5. All constructs were verified by DNA sequencing.

### Histochemical staining

Lignin was detected using Wiesner reagent (3% [v/v] phloroglucinol-HCl)^73^. Five days after nematode inoculation, galls, and root tips were harvested, embedded in 7% (w/v) agarose, and cut into 50-μm sections with a vibratome (Leica VT 1000 S, Germany). Sections were incubated for 5 min in Wiesner reagent, which was subsequently replaced by sterile water. A light microscope (Leica DM2500, Germany) was used for the imaging. Callose staining was performed according to Millet et al.^74^. Three days after nematode inoculation, ~2-cm-long roots tips were cut and immediately fixed in a 3:1 (v/v) ethanol:acetic acid solution for 24 h. After rehydration and water washes, roots were treated with 10% (w/v) NaOH at 37°C for 2 h to clear the tissues. Then, the roots were incubated in 0.01% (w/v) aniline blue in 150 mM K_2_HPO_4_ solution (pH 9.5) for 25 min. The roots, including galls, were mounted onto slides, and callose deposition was examined immediately using a microscope under UV light (excitation, 390 nm; emission, 460 nm). For *MG1* promoter-*GUS* analysis, root fragments with or without nematode infection were vacuum-infiltrated with GUS staining solution (100 mM Tris-HCl, pH 7.0, 50 mM NaCl, 1 mM 5-bromo-4-chloro-3-indolyl-β-glucuronic acid, 1 mM potassium ferricyanide, pH 7.0, and 0.06% [v/v] Triton X-100) twice for 10 min each and incubated overnight at 37 °C. The reaction was stopped with 70% (v/v) ethanol. Stained roots were visualized with a stereoscope.

### Agroinfiltration assays in *N. benthamiana*

*A. tumefaciens* strain EHA105 harboring different binary vectors was syringe-infiltrated into fully expanded leaves of 5-week-old *N. benthamiana*. Briefly, the *A. tumefaciens* cultures were pelleted and resuspended in infiltration buffer (10 mM MES, pH 5.6, 10 mM MgCl_2_, and 200 μM acetosyringone) to a final concentration of OD_600_ = 1.0. To enhance transient gene expression, an *A. tumefaciens* strain carrying the p19 silencing suppressor was included with all infiltrations. The suspensions were kept at 25 °C for at least 3 h without shaking. For co-infiltration, equal volumes of suspensions carrying the indicated constructs were mixed and infiltrated into *N. benthamiana* leaves. After infiltration, plants were cultured at 25 °C for 36–48 h in a 16-h-light/8-h-dark photoperiod with a light intensity around 100 μmol m^−2^ s^−1^. Images presented in the figures are representative of at least five leaves. For cell death analysis, leaves were exposed under UV light (365 nm) using an ultraviolet imager.

### Subcellular localization assay

*MG1* was transiently transfected in rice protoplasts to investigate MG1 subcellular localization. Rice protoplasts from 10-day-old ZH11 seedlings were isolated and transfected^75^. In brief, rice sheath tissues were cut into 0.5 mm strips and subjected to enzymatic digestion in solution containing 1.5% cellulase R10 and 0.75% Macerozyme R10 for 5.5 h. Then the protoplasts were washed with W5 solution (5 mM KCl, 154 mM NaCl, 125 mM CaCl_2_, and 2 mM MES, pH 5.7), and re-suspended in MMG solution (0.6 M Mannitol, 4 mM MES, and 15 mM MgCl_2_) to a final concentration of 1.0 × 10^7^ ml^–1^. For transfection, 10 µl of plasmids (5–10 µg) was mixed with 100 µl of protoplasts and 110 µl of PEG–CaCl_2_ solution (100 mM CaCl_2_, 0.6 M Mannitol, and 40% PEG4000). Transfected protoplasts were incubated in W5 solution for 16 h and used for fluorescence observation. To confirm MG1 subcellular localization, the *MG1-GFP* construct was transiently infiltrated in *N. benthamiana* leaves as described above. Fluorescence signals were captured with a Leica TCS SP8 confocal microscope system using an excitation laser wavelength of 488 nm with an emission filter of 500–550 nm.

### Y2H assay

The Y2H screening was conducted using the LRR or CC domain of MG1 as the bait following the manufacturer’s instructions (Matchmaker Gold Yeast Two-Hybrid Library Screening System, Clontech, Dalian, China). A root cDNA library of non-inoculated rice cultivar ZH11 generated with Make Your Own ‘Mate & Plate’ Library System (Clontech, Dalian, China) was used for screening. Positive protein–protein interactions were selected by growing yeast colonies on synthetic defined (SD) medium lacking leucine, tryptophan, and histidine (SD–L–T–H, TDO). Combinations of AD/SV40 with BD/p53 or BD/Lam served as positive or negative controls, respectively. To test MG1 self-interaction and to confirm the protein–protein interactions, different combinations of *MG1* fragments and their interacting candidates, as indicated in the figures, were co-transformed into yeast strain AH109. Colonies grown on SD–L–T (DDO) medium were cultured, diluted, and plated on TDO medium. Plates were kept at 30 °C for 3 d before being photographed.

### Firefly LCI assay

To investigate the protein interactions, the LCI assays were carried out as previously described^76^. *A. tumefaciens* strain EHA105 carrying the indicated constructs was co-infiltrated into *N. benthamiana* leaves as described above. Two days after infiltration, the infiltrated leaves were sprayed with 1 mM luciferin (Sigma-Aldrich, L9504) supplemented with 0.01% (v/v) Triton X-100 in darkness for 3 min. Luminescence images were captured using a low-light cooled CCD imaging apparatus (NightOWL II LB983).

### Co-IP assay

*A. tumefaciens*–infiltrated leaves were collected, and total protein was extracted by incubating the homogenized samples in protein extraction buffer (10% [v/v] glycerol, 20 mM Tris-HCl, pH 7.5, 1 mM EDTA, 100 mM NaCl, 0.5% [v/v] Triton X-100, and 1× protease inhibitor cocktail from Roche, USA) for 30 min. After centrifugation at 10,000 × *g* for 10 min, the supernatant was incubated with anti-GFP agarose beads (Chromotek, gta-20) for 3 h at 4 °C. The beads were washed four times with extraction buffer. The proteins were then eluted by boiling with 2× SDS loading buffer for 5 min. Co-immunoprecipitated proteins were analyzed by immunoblot with 1:5000 diluted anti-FLAG, anti-GFP and anti-Actin antibodies (Sigma-Aldrich and ABclonal). The Clarity Western ECl Substrate (Bio-Rad) and Odyssey LI-COR Imaging System were used to detect the blot signals.

### Chemical treatments

To inhibit JA biosynthesis, 100 μM diethyldithiocarbamic acid (DIECA), purchased from Sigma, was exogenously applied to rice leaves 24 h before nematode inoculation. DIECA was prepared in water containing 0.02% (v/v) Tween 20. Distilled water containing 0.02% (v/v) Tween 20 was used as a control. For the cell death assay, the palmitoylation inhibitor 2-bromopalmitate (2-BP) was infiltrated at a concentration of 100 μM into *N. benthamiana* leaves 6 h before *MG1* agro-infiltration. To determine MG1 protein stability, 50 μM cycloheximide (CHX) was infiltrated into *N. benthamiana* leaves 4 h before immunoblot analysis.

### Statistical analysis

The Chi-square test for goodness-of-fit was performed with MS Excel. The nematode infection and gene expression data were analyzed by GraphPad Prism 6 software. Significant differences between the treatments were evaluated using one-way analysis of variance (ANOVA) analysis followed by Fisher’s LSD multiple comparison test at a 5% probability level.

### Reporting summary

Further information on research design is available in the Nature Portfolio Reporting Summary linked to this article.

## Supplementary information

Supplementary Information

Reporting Summary

## Data Availability

The RNA-seq and QTL-seq data are available in the NCBI database with accession numbers PRJNA907725 and PRJNA907931. The data supporting the findings of the study are available from the corresponding author upon request. Source data are provided with this paper.

## Source data

Source Data
