# Supplementary material for: MG1 interacts with a protease inhibitor and confers resistance to rice root-knot nematode
Source: Nat Commun. 2023 Jun 8;14:3354. doi: 10.1038/s41467-023-39080-6 (PMC10250356; doi:10.1038/s41467-023-39080-6)
Supplement: Supplementary file 1 — Supplementary Information [file 41467_2023_39080_MOESM1_ESM.pdf]

Supplementary Figures and Tables

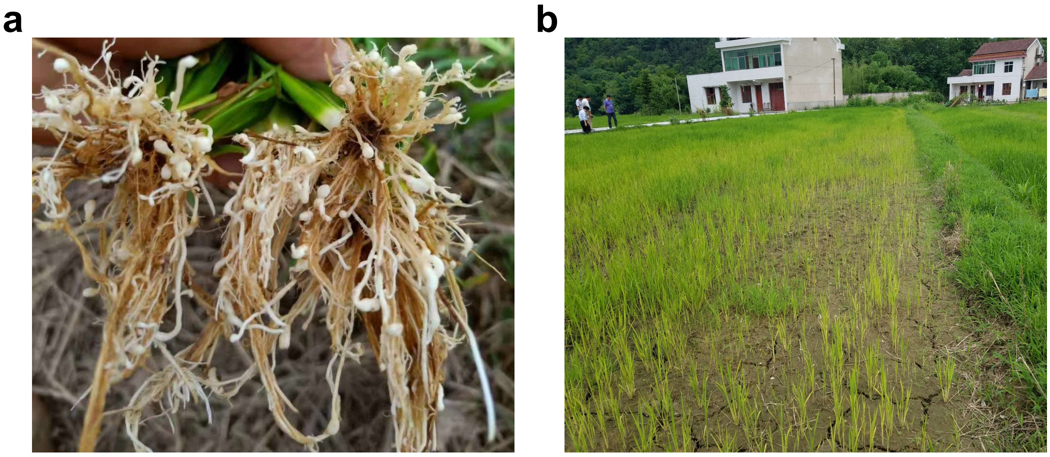

Supplementary Figure 1. Rice symptoms in field infested with *M. graminicola*. **a** Typical hook-shaped galls at rice root tips. **b** Seedling death and patchy chlorosis distribution in upland rice field in Hunan province, China.

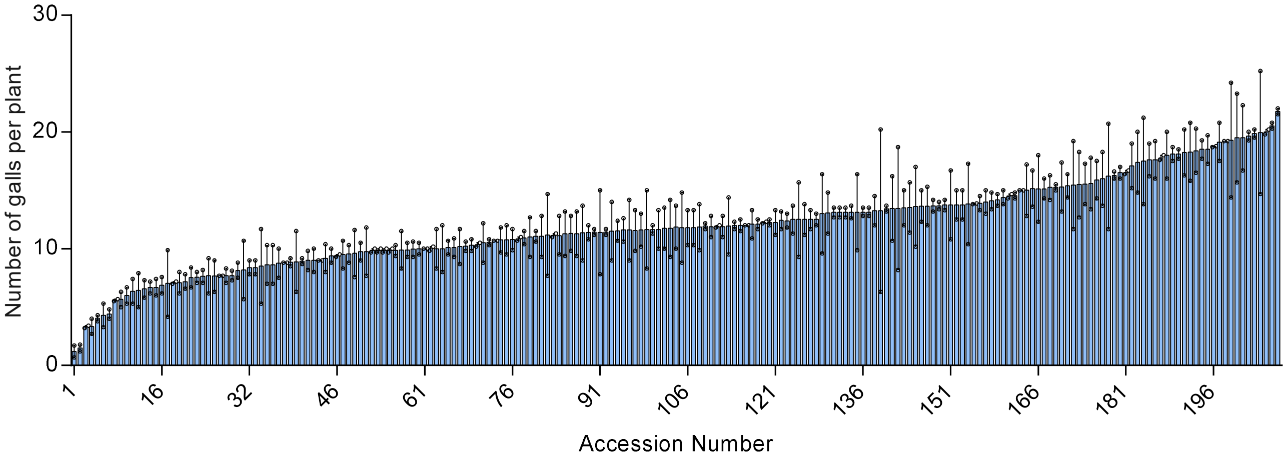

Supplementary Figure 2. Number of galls on rice varieties after inoculation with *M. graminicola*. Data were collected from twelve plants of each rice variety at two weeks after inoculation with 150 J2s. Data are means ± s.e.m. (n = 2 independent replicates). Related data were listed in Supplementary Table 1.

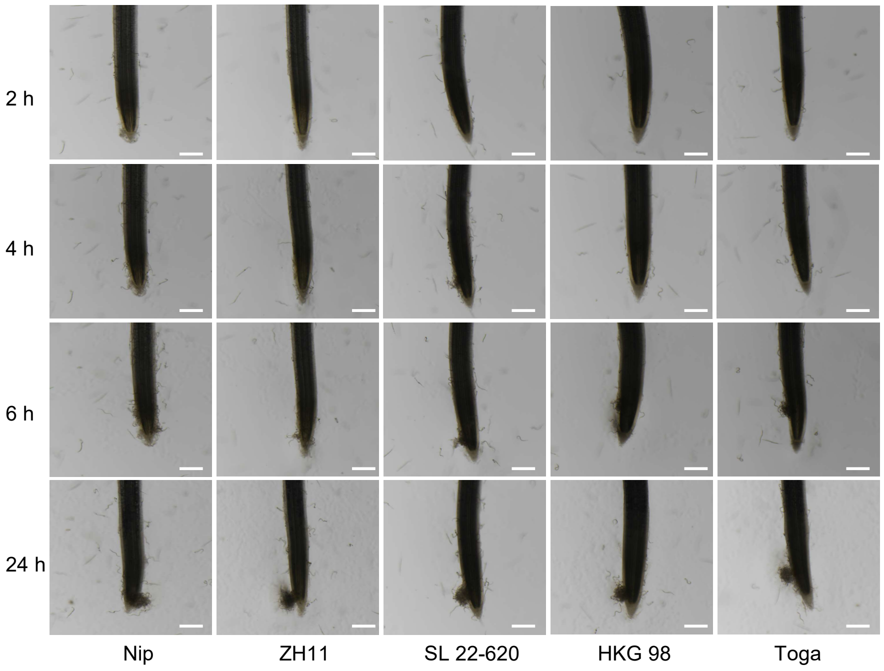

Supplementary Figure 3. Attraction of *M. graminicola* to the susceptible and resistant rice varieties at the indicated time points. Representative image showing the nematode attraction at the root tips in pluronic F-127 gel containing 1000 J2s. Scale bar, 500 μm. The experiments were performed three times with similar results.

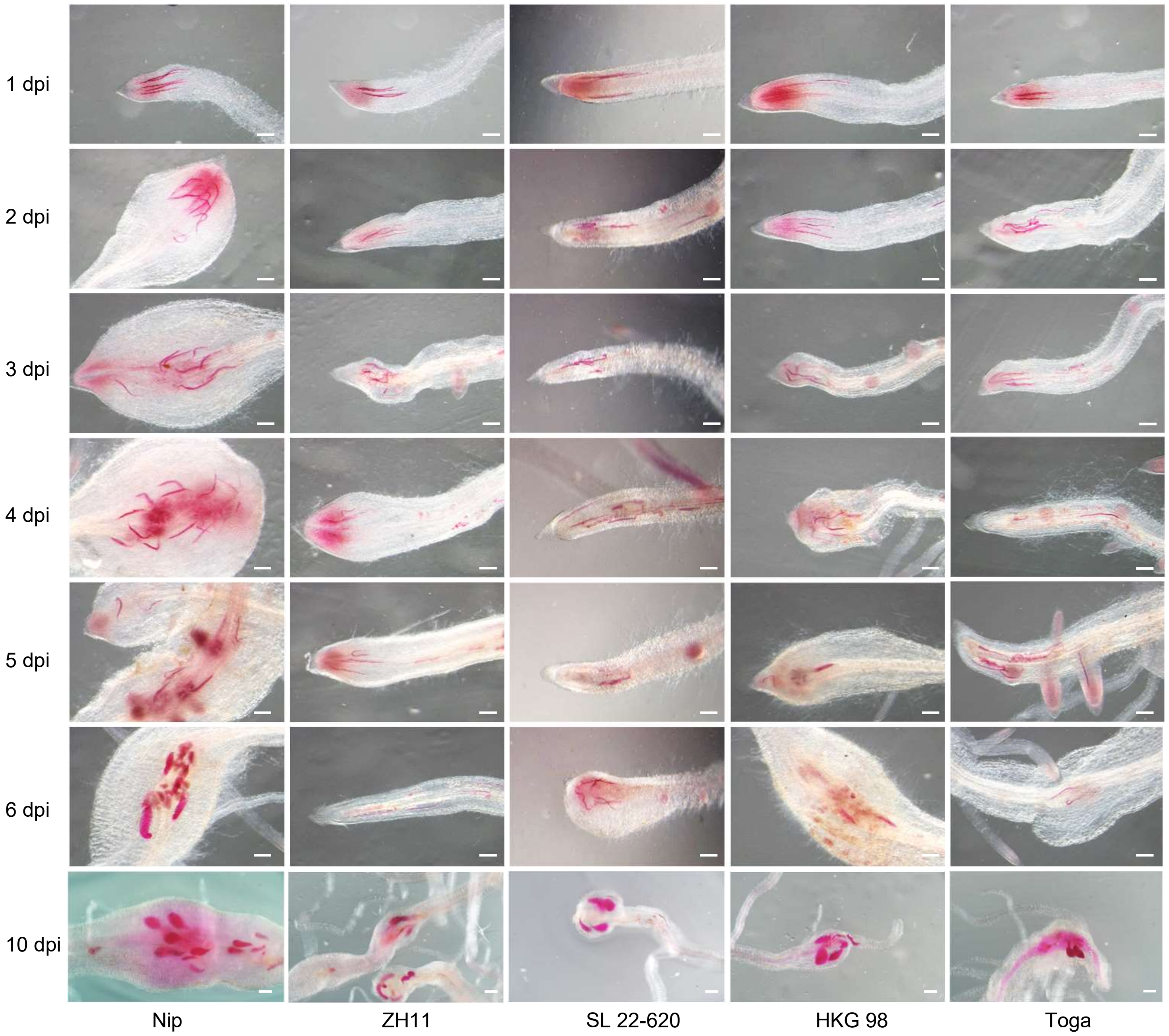

Supplementary Figure 4. Comparison of nematode development in different rice varieties at the indicated time points. Nematodes were visualized with acid fuchsin staining. Scale bar, 200 μm. The experiments were performed three times with similar results.

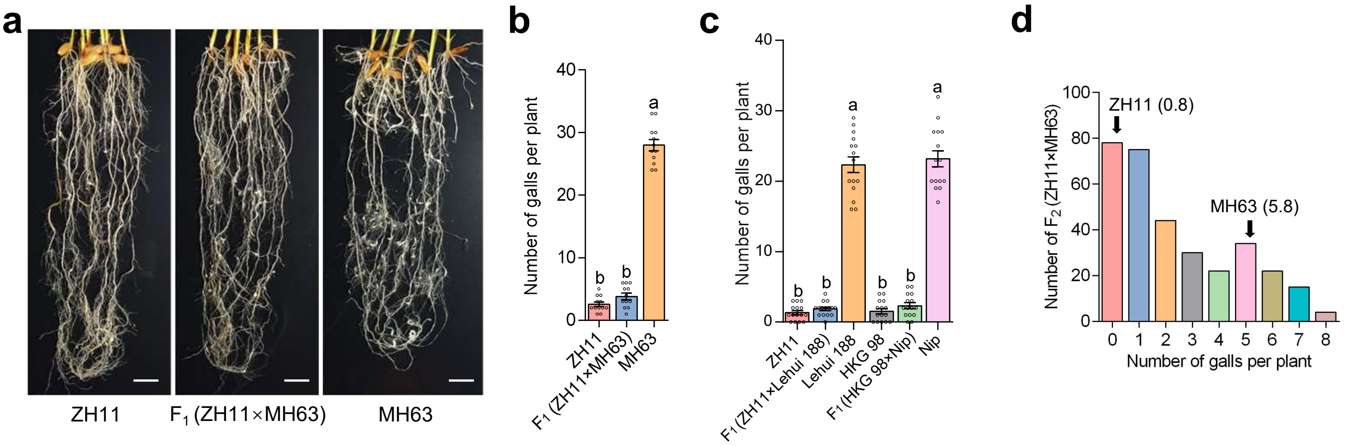

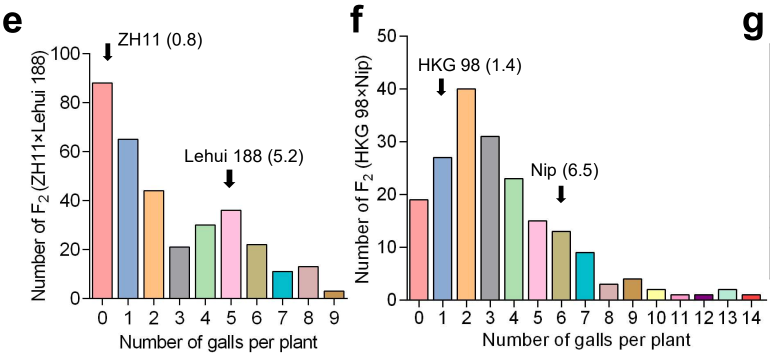

g

Generation	Total plants	R:S ratio		χ^2（3:1）	*P*
		Observed	Expected		
F_2（ZH11×Lehui 188)	333	248:85	250:83	0.036<3.84	＞0.05
F_2（ZH11×MH63)	324	249:75	243:81	0.498<3.84	＞0.05
F_2（HKG 98×Nip)	191	155:36	143:48	3.534<3.84	＞0.05

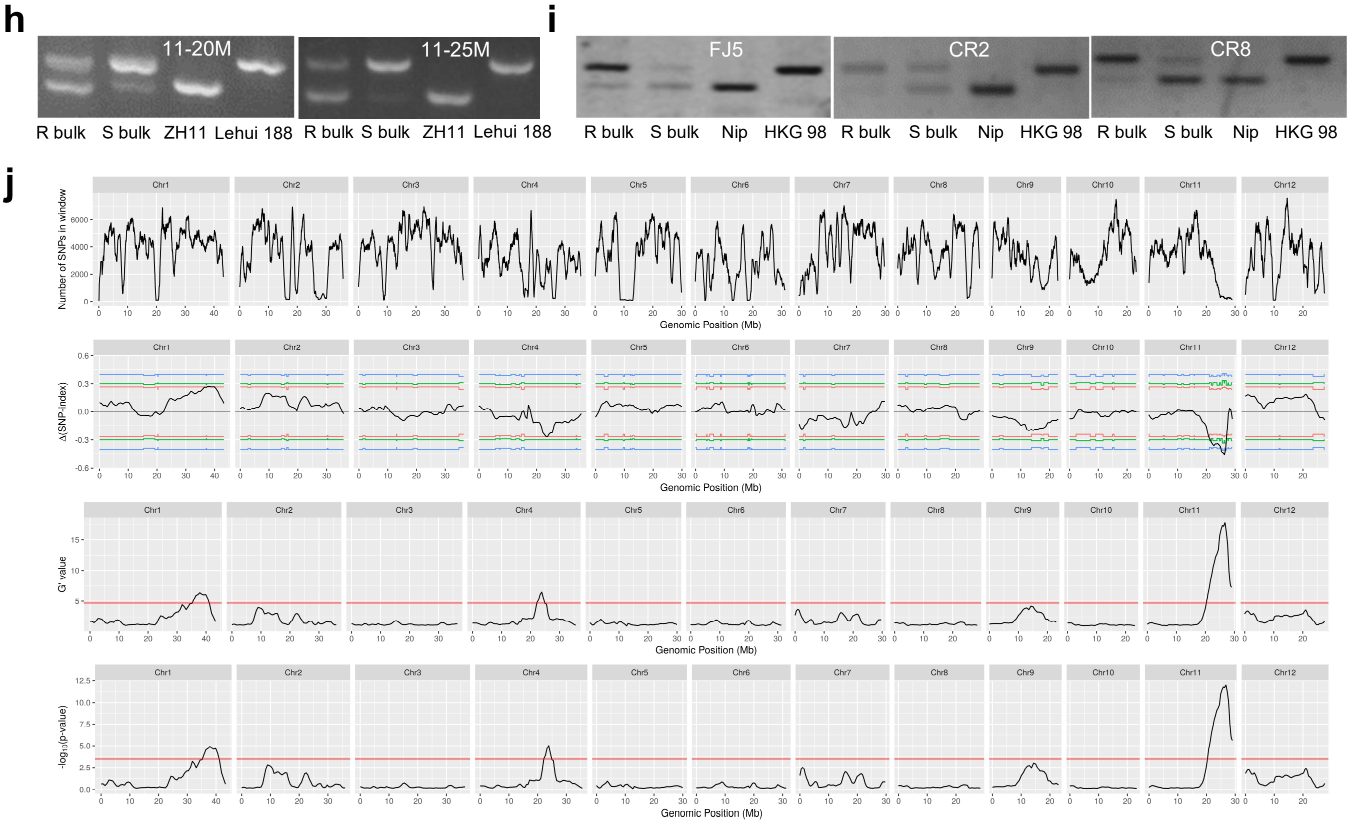

Supplementary Figure 5. Map-based cloning of *MG1* in ZH11. a Representative root disease symptoms of F_1 plants derived from the cross between ZH11 and MH63 after *M. graminicola* infection. Scale bar, 2 cm. **b, c** Number of galls on F_1 plants at 15 days after inoculation with 150 J2s of *M. graminicola*. Data are the means ± s.e.m. from one representative experiment (*n* = 12 independent plants in **b**, *n*=15 independent plants in **c**). **d-f** The frequency distribution of gall numbers in F_2 plants of three populations (ZH11×Lehui 188, ZH11×MH63, and HKG 98×Nip). The arrows denote average gall number of the parents. **g** The segregation of the resistant to susceptible plants in F_2 populations fits well to a 3:1 ratio. Data were collected at 15 days after infection with *M. graminicola*. S, susceptible; R, resistant. Chi-square test values are provided. **h** Two polymorphic markers distinguishing R and S pools from ZH11×Lehui 188. **i** Three polymorphic markers distinguishing R and S pools from HKG 98×Nip. **j** QTL-seq results for all chromosomes. The x-axis corresponds to the chromosomal position. The Δ(SNP-index) plot (the 2nd top) is shown with statistical confidence intervals under the null hypothesis of no QTL (gray, $P < 0.1$; green, $P < 0.05$; pink, $P < 0.01$). For G' value and its corresponding *P* value ($-\text{Log}_{10}$ value) plot (bottom two), red threshold line represents $q < 0.01$. Different letters above the bars in (**b**, **c**) indicate statistical significance groups at $P < 0.05$ (one-way ANOVA analysis followed by Fisher's LSD multiple comparison test). Exact *P* values are provided in the Source Data file (**b**, **c**).

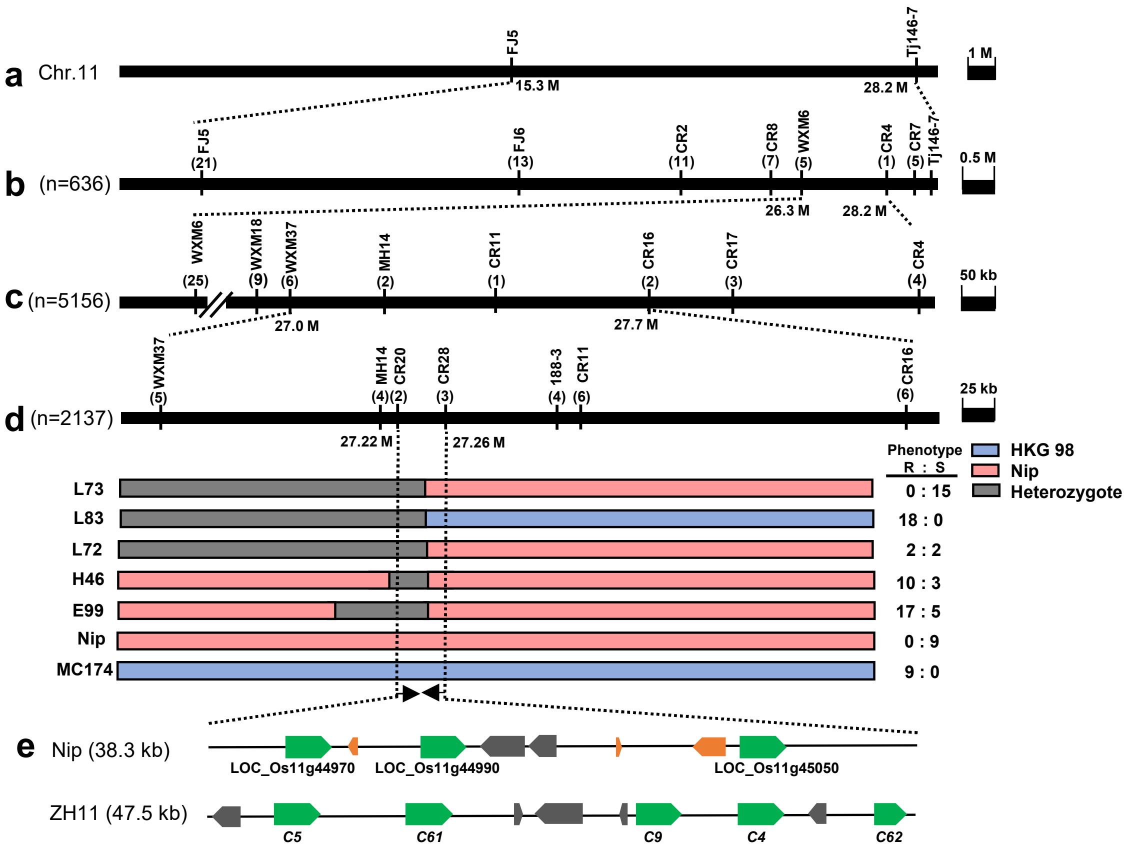

Supplementary Figure 6. Fine mapping of *MG1* in HKG 98. a *MG1* was located between FJ5 and Tj146-7 on chromosome 11. **b-d** *MG1* was fine mapped to a region flanked by markers CR20 and CR28 using F_2 and $F_{2:3}$ population. Recombinant number detected between the molecular markers are indicated in the bracket below the markers. The numbers below the linkage map represent the location of the markers. Genotypes and phenotypes of five recombinants are included in (**d**). Different color boxes denote the marker genotypes. **e** The predicted ORFs within the mapping intervals of ZH11 and Nipponbare. Green arrows indicate the predicted *NLR*s and orange arrows represent expressed genes. The transposable elements are indicated in gray.

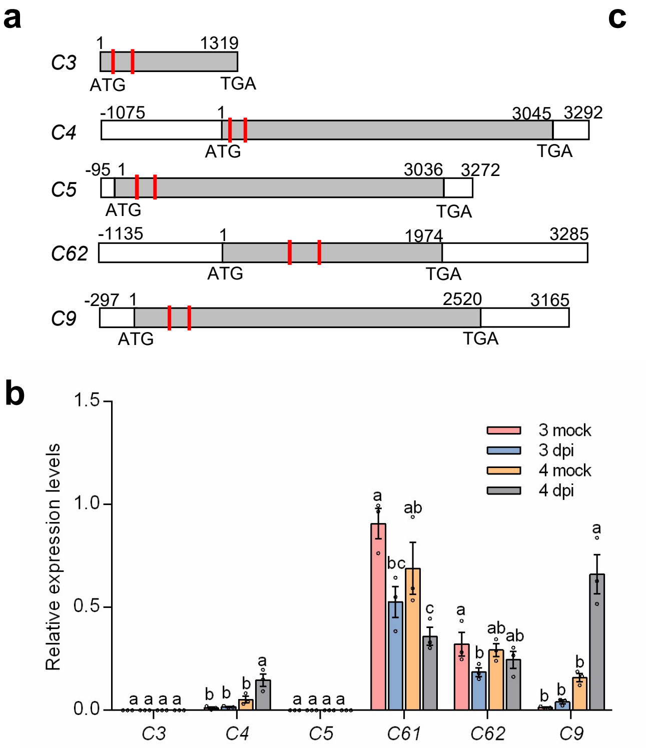

c

	gRNA1	gRNA2
C3 WT	TAGAACCCAACAAAATCCACTGG	GTTTGCCTAGTGCAATCACTCGG
C3-10	TAGAACCCAACAAAATCCACTGG	GTTTGCCTAGTGCAATCAACTCGG
C4 WT	GTATTAGTGATGCTGAGGCAAGG	ATCATCCTTCATCGTCATCAAGG
C4-37	GTATTAGTGATGCTGAGTGCAAGG	ATCATCCTTCATCG - - -TCAAGG
C4-55	GTATTAGTGATGCTGA- GCAAGG	ATCATCCTTCATCGTC -TCAAGG
C5 WT	GTATTAGTGATGCTGAGGCAAGG	ATCATCCTTCATCGTCATCAAGG
C5-29	GTATTAGTGATGCTGA- GCAAGG	ATCATCCTTCATCGT - - TCAAGG
C5-35	GTATTAGTGATGCT - - - GCAAGG	ATCATCCTTCATCGTC - TCAAGG
C62 WT	CTCAGCCGCACACAACATAATGG	TGCCTGGGTTTGTGTCTCCAAGG
C62-15	CTCAGCCGCACACAACA - - - - - - - - - - - 248bp - - - - - - - - - - - - CCAAGG	
C62-49	CTCAGCCGCACACAA - -TAATGG	TGCCTGGGTTTGTG - - TCCAAGG
C9 WT	TTCTAGAGATTTGGTGAGGCAGG	GAAGCTTCAGCAAACATGCATGG
C9-6	TTCTAGAGATTTGGTGATGGCAGG	GAAGCTTCAGC - - - - - - GCATGG
C9-44	TTCTAGAGATTTGGTGATGGCAGG	GAAGCTTCAGCAAAC- - GCATGG

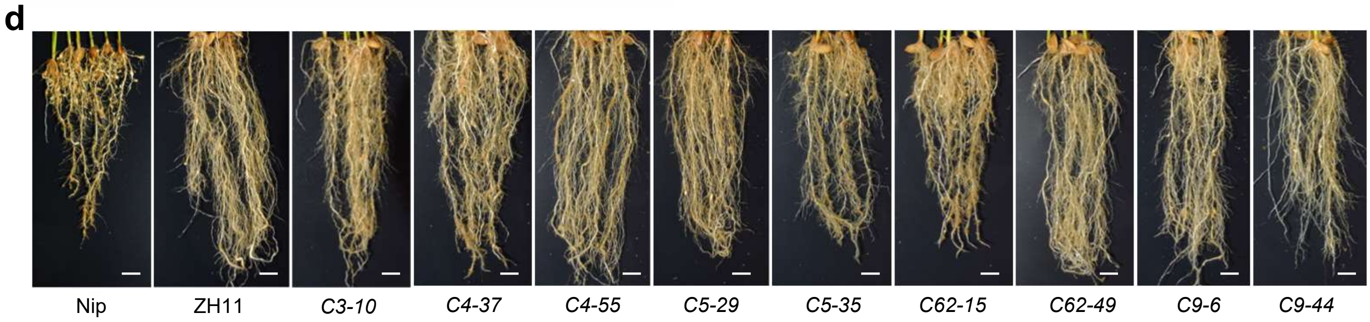

Supplementary Figure 7. CRISPR/Cas9-mediated knockout of candidate genes in ZH11. a Diagram showing gene structure and two editing sites of the candidate genes. Exons are indicated in gray and untranslated regions are in white. **b** The expression level of candidate genes in root tips of ZH11. qRT-PCR analysis was performed at 3 and 4 days after nematode infection. *OsEXPNAR* was used as an internal control. Data are means ± s.e.m. of three independent biological replicates. Different letters above the bars indicate statistical significance groups at $P < 0.05$ (one-way ANOVA analysis followed by Fisher's LSD multiple comparison test). **c** Sequencing results of the two targeting sites in candidate genes. The insertions are shown in red color, and short lines indicate deletions. **d** Representative root symptoms of CRISPR-edited mutants at 15 days after *M. graminicola* infection. Scale bar, 2 cm. The experiments were performed three times with similar results.

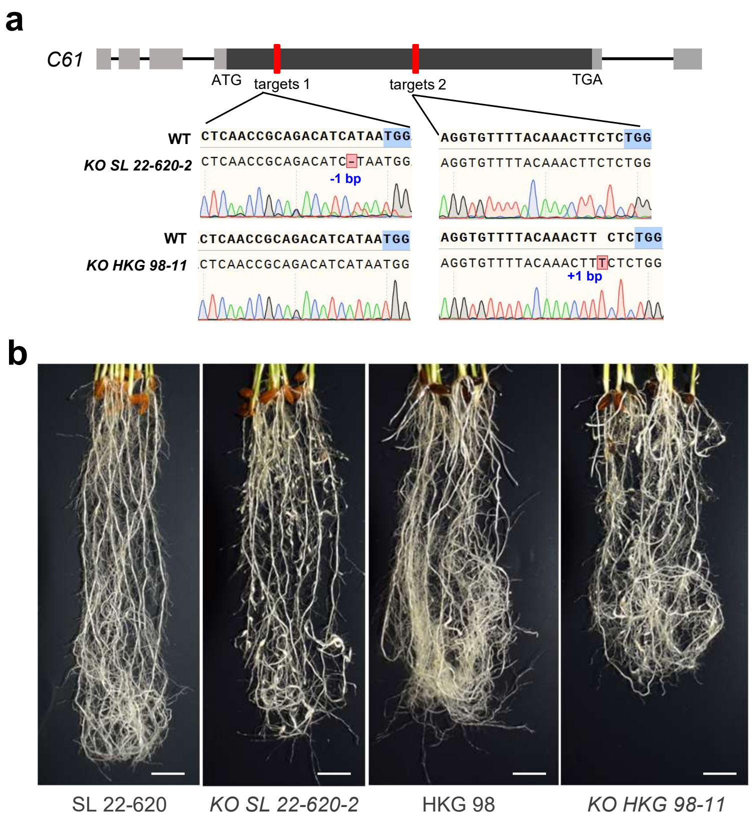

Supplementary Figure 8. CRISPR/Cas9-mediated knockout of *MG1* in SL 22-620 and HKG 98. a The sequencing results showing the mutations sites of *MG1* in SL 22-620 and HKG 98 background. Short line indicate deletion and red letter indicate insertion. **b** Root disease symptoms of CRISPR-edited *MG1* mutants after *M. graminicola* infection. Scale bars, 2 cm.

a

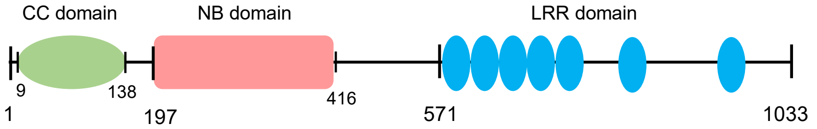

b

Coiled-coil

MATIVDTLVGSCINKLQAIITDKTILILGVKDELEELQRRTNVIRSSLQDAEARRMEDSVVEK
WLDQLRDVMYDVDDIIDLARFKGSVLLPDYPMSSSRKSTACSGLSLSSCFSNIRIRHEVAVKI
RSLNKKIDNISKDEVFLKLNRRHHNGSGSAWTPIESSSLVEPNLVGKEVIRACREVVDLVLA
HKKKNVYKL

NB

AIVGTGGVGKTTLAQKIFNDKKLEGRFDHHAWACVSKEYSRDSLLRQVLRNMGIRYEQD
P-loop RNBS-A

ESVPELQRKIKSHIANKSFFLVLDDVWNSEAWTDLLSTPLHAAATGVILITTRDDTIARVIG
Kinase 2 RNBS-B

VDHTHRVDLMSADVGWELLWRSMNIKEEKQVKNLRDTGIEIVRKCGGLPLAIRAIAKVL
GLPL

ASLQDQTENEWRQILGKNAWSMSKLPDELNGALYLSYEVLPHQLKQCFLYCALFPEDATI
RNBS-D

FCGDLTRMWVAEGFIDEQEGQLLEDTAERYYHELIHRNLLQPDGLYFDHSRCKMHDLLRQ
RNBS-D MHD

LASYLSREECFVGDPESLGTNTMCKVRRISVVTEKDIVVLPSMDKDQYKVRCFTNFSGKSA

RIDNSLFKRLVC

LRR

LRILDLAGSLVHDIPGAIGNLIY
LRLLDLDRTNICSLPEAIGSLQS
LQILNLQGCES
LRRLPLATTQLCN
LRRLGLAGTPINQVPKGIGRLKFLNDLEGFPIGGGNDNTKIQDGWNLEELGHLSQLRCLDMI
KLERATPCSSTDPFLLSEKKH
LKVLNLHCTEQTDEAYSEEGISNVEKIFEKLEPPHNLEDLVIGDFFGRRFPTWLGSTHLSSVK
YVLLIDCKSCVHLPPIGQLPNLKYLKIIGASAITKIGPEFVGCWEGNLRSTEAVAFPKLEMLIF
KEMPNWEEWSFVEEEEVQEEAAAAAKEGGEDGIAASKQKGEEAPSPTPRSSWLLPC
LNELQLVNCPKLRALPPQLGQQATNLNEILIRDTRYLKTVEDLPFLSGFLQVEGCEGLERVS
NLPQVRELFVNECPNLRHVEELGGLEQLLLDEGMQEISSLWVPRLQEQHRQLHGDEHELEV
IEWL

Supplementary Figure 9. Sequence analysis of MG1 protein. a Schematic representation of domain structure of MG1. **b** Amino acid sequence of the MG1. The conserved motifs (P-loop, RNBS-A, Kinase2, RNBS-B, GLPL, RNBS-D and MHD) are labeled in red. The C-terminal leucine-rich repeat (LRR) domain consists of 7 imperfect LRR repeats predicted by the LRR search programs (https://lrrsearch.com/).

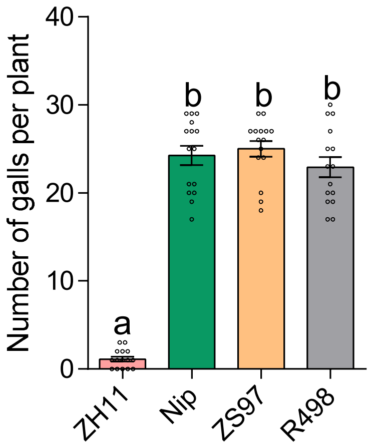

Supplementary Figure 10. Nematode susceptible phenotype of ZS97 and R498 at 15 dpi. Data are means ± s.e.m from one representative experiment (*n* = 15 independent plants). Different letters above the bars indicate statistical significance groups at $P < 0.05$ (one-way ANOVA analysis followed by Fisher's LSD multiple comparison test). Exact *P* values are provided in the Source Data file. The experiments were repeated two times with similar results.

a

Marker	Primer sequence （5'- 3'）	Marker type	Expected size (bp)	Cutting enzyme
CR24	F: TCGTCATGTGGTTTCTGGC	Indel	423	
	R: TGTTGCAGTTGCTTCTGC			
WXM1	F: GAGGAGTGGTCCTTTGTTGAAG	CAPS	421	MboI
	R: ATAGCTGCTCCAAACCACCCAAC			

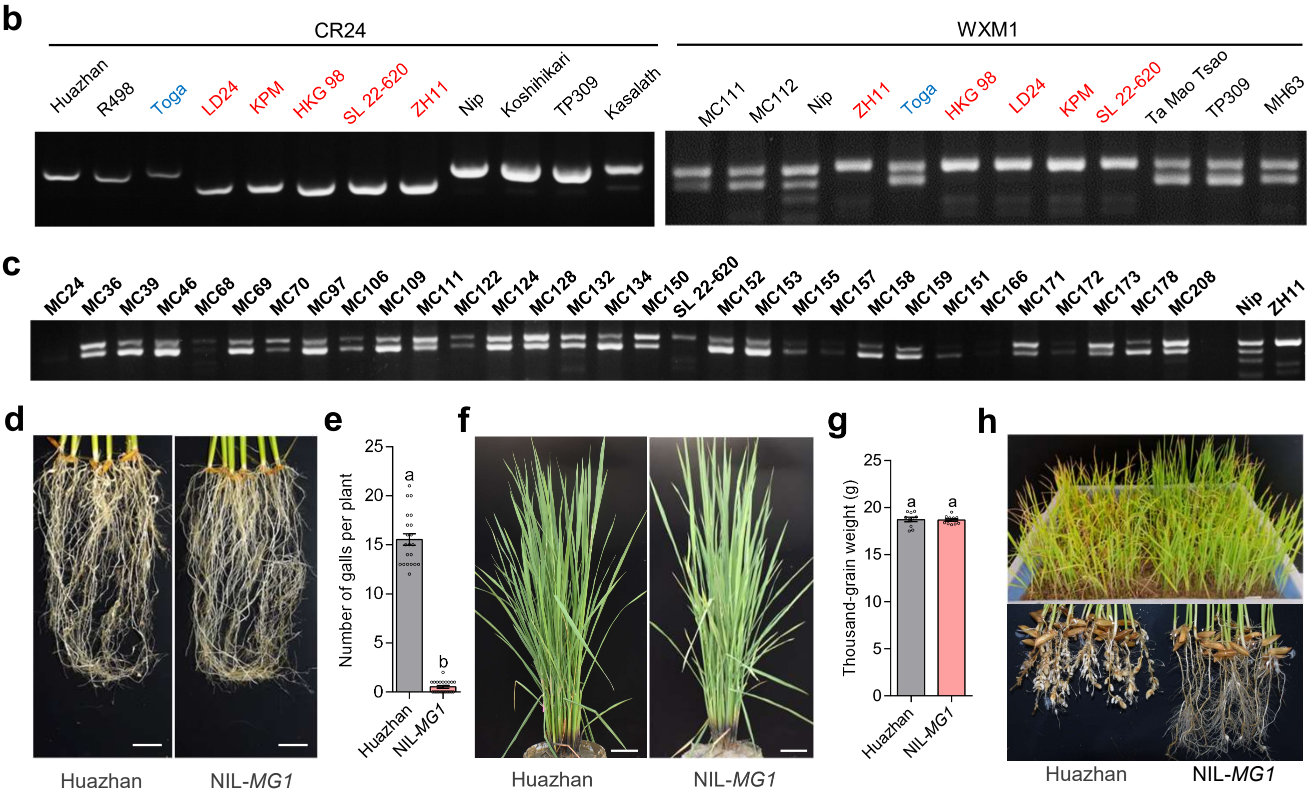

Supplementary Figure 11. The introgression of MG1 in rice breeding. a Information on the *MG1*-linked molecular markers. Two markers (WXM1 and CR24) were developed for screening. **b** PCR assay of different varieties using the molecular markers WXM1 and CR24. **c** PCR assay of different varieties using WXM1. All the rice varieties belong to *aus* subpopulation. **d** Root disease symptoms of near isogenic lines containing *MG1* after nematode infection. **e** Number of galls of NIL-*MG1* at 15 days after nematode infection. Data are means ± s.e.m from one representative experiment (n = 21 independent plants). **f** Morphology of NIL-*MG1* grown in the field. **g** Thousand-grain weight (g) of NIL-*MG1*. Data are means ± s.e.m. from one representative experiment (n = 10 independent plants). **h** Growth phenotype of NIL-*MG1* in nematode-infested soil under greenhouse conditions. Scale bars, 2 cm (**d**), 10 cm (**f**). Different letters above the bars in (**e**, **g**) indicate statistical significance groups at $P < 0.05$ (one-way ANOVA analysis followed by Fisher's LSD multiple comparison test). Exact P values are provided in the Source Data file. The experiments were performed three times with similar results (**d-h**).

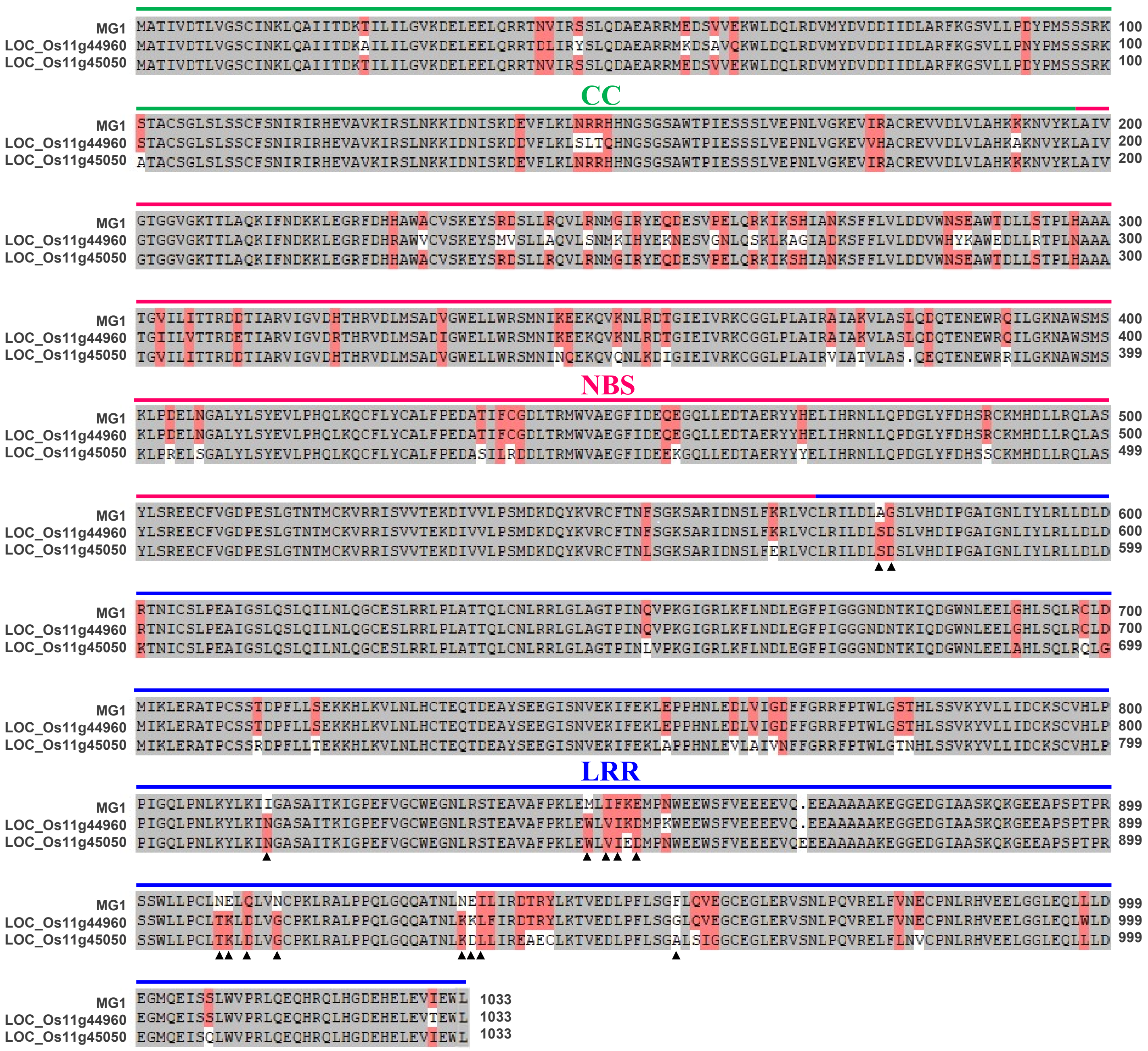

Supplementary Figure 12. Sequence alignment of MG1 and its orthologs in Nipponbare. The protein sequence of MG1, LOC_Os11g44960 and LOC_Os11g45050 were aligned with ClustalX v2.1. The conserved domains were marked above the alignment. Black triangles indicate fifteen unique amino acid substitutions in MG1.

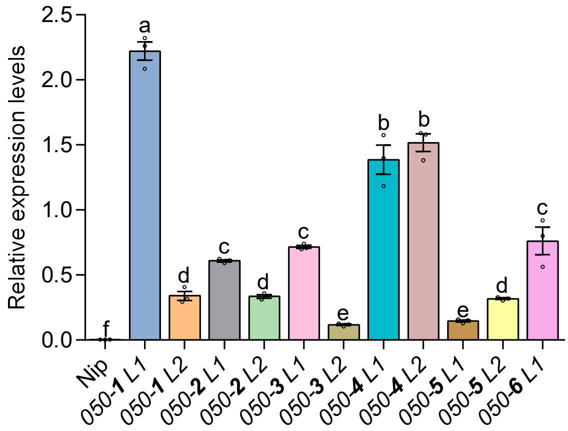

Supplementary Figure 13. Detection of expression level for different transgenes using qRT-PCR. Bars are means ± s.e.m. of three independent biological replicates with two technical replicates. Different letters above the bars indicate statistical significance groups at $P < 0.05$ (one-way ANOVA analysis followed by Fisher's LSD multiple comparison test). Exact *P* values are provided in the Source Data file.

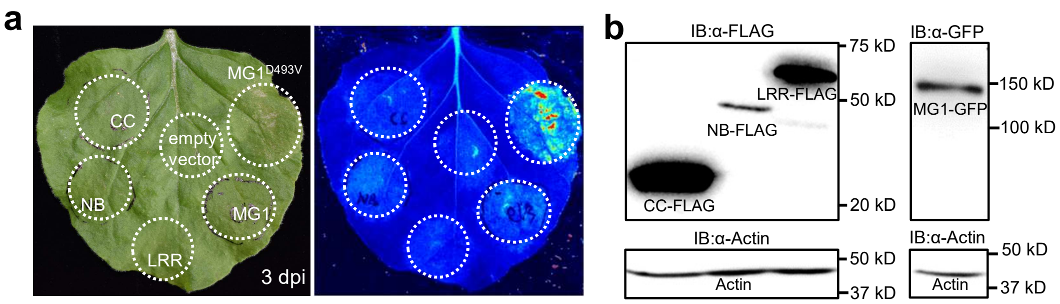

Supplementary Figure 14. Observation of MG1-mediated cell death phenotypes. **a** CC, NB, and LRR domain of MG1 and MG1^{D493V} were transiently expressed in *N. benthamiana*. **b** Detection of protein expression by immunoblotting with anti-FLAG and anti-GFP antibodies. Leaves were photographed at 3 days after infiltration. The protein loading is shown by an anti-Actin immunoblot. The experiments were repeated three times with similar results.

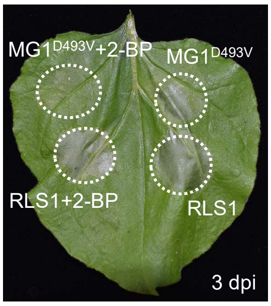

Supplementary Figure 15. The effect of 2-BP treatment on the cell death phenotype induced by *MG1^{D493V}* or *RLS1* in *N. benthamiana* leaves. The experiments were repeated three times with similar results.

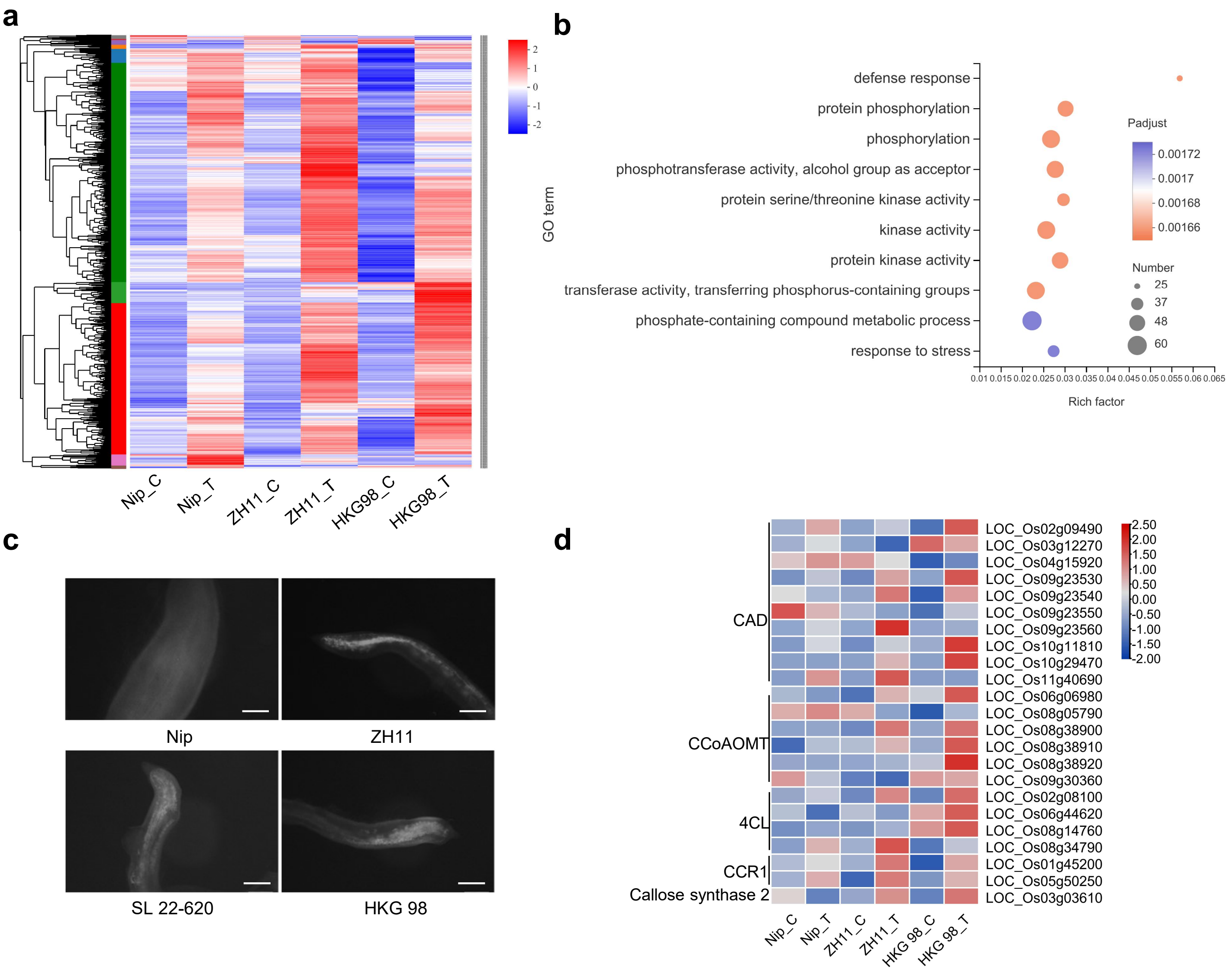

Supplementary Figure 16. RNA-seq analysis of different rice varieties. a Heatmap analysis of the expression patterns of all DEGs in susceptible (Nip) and resistant (ZH11 and HKG 98) rice varieties. Heat map is arranged according to their hierarchical clustering of gene expression pattern. The colors represent upregulation (red) and downregulation (blue) with $Log_{10}(TPM+1)$ values. Nip_C, ZH11_C and HKG 98_C represent control samples. Nip_T, ZH11_T and HKG 98_T represent nematode-treated samples. **b** GO analysis of 339 DEGs co-upregulated in ZH11 and HKG 98. **c** Callose staining of different rice varieties. Samples were taken for photograph 3 days after inoculation with 150 J2s. Scale bar, 500 μm. The experiments were repeated three times with similar results. **d** Expression analysis of genes related to lignin and callose synthesis in resistant and susceptible varieties.

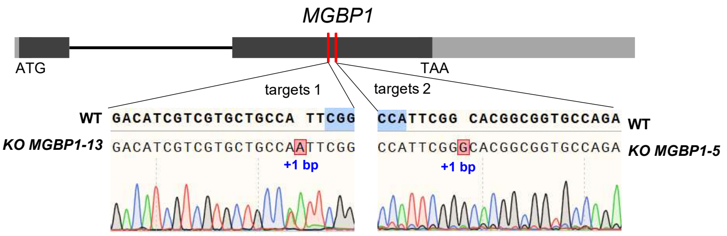

Supplementary Figure 17. The CRISPR/Cas9 editing sites of *MGBP1* in ZH11. Sequences of target sites in the wild type and two homozygous mutants (*MGBP1-5* and *MGBP1-13*) are aligned. The protospacer adjacent motif (PAM) is marked with blue background.

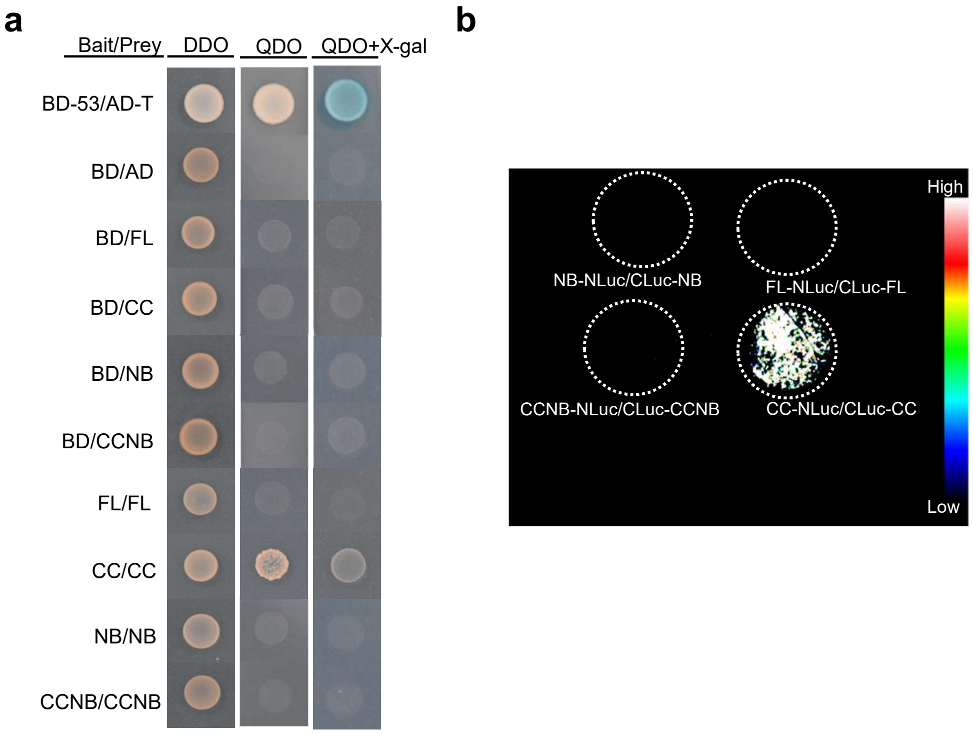

Supplementary Figure 18. Detection of self-association of MG1. The indicated constructs containing full length and different domains of MG1 were used for Y2H (**a**) and split luciferase assays in *N. benthamiana* (**b**). The experiments were repeated three times with similar results.

Supplementary Table 1. Number of galls on different rice varieties after inoculation with *M. graminicola* .

Accession ID	Accession number	Accession Name	Origin	Species	Number of galls (1)	Number of galls (2)	Average
MC79	1	Toga	India	IND	1.7	0.7	1.2
ZH11	2	ZH11	China	TEJ	1.8	1.2	1.5
MC174	3	HKG 98	Mali	AUS	3.4	3.2	3.3
MC2	4	Mayang Khang	Indonesia	IND	4.0	2.7	3.3
MC162	5	SL 22-620	Sierra Leone	AUS	4.3	3.8	4.1
MC175	6	Daudzai Field Mix	Pakistan	AUS	3.3	5.3	4.3
MC204	7	Bogarigbeli	Burkina Faso	IND	4.0	4.8	4.4
MC34	8	Warrangal Culture 1252	India	IND	5.5	5.7	5.6
MC196	9	Nahng Sawn	Thailand	IND	5.0	6.3	5.7
MC195	10	A 36-3	Myanmar	IND	6.7	5.3	6.0
MC177	11	Karabaschak	Bulgaria	TEJ	7.4	5.3	6.4
TP309	12	TP309	China	TEJ	5.0	7.9	6.5
MC4	13	RD 218	Dominican Republic	Admix(TEJ-TRJ)	7.3	5.8	6.6
MC118	14	IR 2061-214-2-3	Philippines	IND	6.2	7.2	6.7
MC5	15	C 5560	Thailand	TRJ	6.0	7.4	6.7
MC36	16	NC 1/536	Pakistan	AUS	6.2	7.6	6.9
MC33	17	C 8429	Papua New Guinea	TRJ	4.2	9.9	7.0
MC28	18	H57-3-1	Argentina	Admix(TEJ-TRJ)	7.2	7.0	7.1
MC120	19	Manga 629	Madagascar	IND	6.2	8.0	7.1
TN1	20	TN1	China	IND	6.6	7.8	7.2
MC176	21	JP 5	Pakistan	Admix(AUS-IND)	8.4	6.7	7.5
MC178	22	Hi Muke	Kazakhstan	AUS	8.0	7.1	7.6
MC157	23	DJ 24	Bangladesh	AUS	8.2	7.1	7.7
MC109	24	ARC 6578	India	AUS	6.2	9.2	7.7
MC212	25	EMBRAPA 1200	Brazil	TRJ	9.0	6.3	7.7
MC203	26	WONG CHIM	Hong Kong	IND	7.7	7.7	7.7
MC165	27	Dhan	Nepal	IND	7.1	8.3	7.7
MC68	28	N-2703	Nepal	AUS	7.3	8.1	7.7
MC217	29	NIPPONBARE	Japan	TEJ	7.5	8.8	8.1
MC51	30	WW 8/2290	Netherlands	Admix(TRJ-ARO-IND)	10.7	5.7	8.2
MC30	31	Khao Phoi	Laos	TRJ	9.0	7.8	8.4
MC40	32	Ai Chueh Ta Pai Ku	Taiwan	IND	9.0	7.8	8.4
MC197	33	ARC 10633	India	IND	5.3	11.7	8.5

MC23	34	Red Khosha Cerma	Afghanistan	ARO	10.3	7.0	8.7
MC202	35	Heo Trang	Vietnam	IND	10.3	7.0	8.7
MC39	36	Doble Carolina	Uruguay	AUS	7.5	10.0	8.8
MC94	37	Ardito	Italy	TEJ	8.8	8.8	8.8
MC21	38	KRASNODARSKIJ 424	Russian Federation	TEJ	8.5	9.2	8.9
MC19	39	Chin Chin	Panama	IND	6.3	11.5	8.9
MC216	40	M202	United States	TEJ	9.2	8.7	8.9
MH63	41	MH63	China	IND	9.8	8.2	9.0
MC32	42	J.P. 5	Australia	TEJ	10.0	8.0	9.0
MC75	43	HB-6-2	Hungary	TEJ	9.0	9.0	9.0
Cheng Hui448	44	Cheng Hui448	China	IND	8.0	10.4	9.2
MC211	45	KRASNODARSKIJ 3352	Russian Federation	TEJ	10.0	8.8	9.4
MC45	46	Acheh	Malaysia	IND	9.5	9.3	9.4
MC156	47	DNJ 179	Bangladesh	AUS	8.3	10.7	9.5
MC35	48	Padi Pohon Batu	Malaysia	TRJ	8.8	10.3	9.6
Koshihikari	49	Koshihikari	Japan	TEJ	7.6	11.6	9.6
MC158	50	DJ 102	Bangladesh	AUS	10.5	9.0	9.8
MC215	51	IR64	Philippines	IND	11.8	7.7	9.8
MC44	52	PD 46	Sri Lanka	IND	9.7	10.0	9.8
MC60	53	Dichroa Alef Uslkij	Kazakhstan	IND	9.7	10.0	9.8
MC198	54	Simpor	Brunei	TRJ	9.7	10.0	9.8
MC200	55	FUJISAKA 5	Japan	IND	10.0	9.7	9.8
MC43	56	TJ	Guyana	IND	10.3	9.4	9.9
MC1	57	Karang Serang	Indonesia	TRJ	11.5	8.3	9.9
MC56	58	MAHSURI	Malaysia	IND	9.3	10.5	9.9
MC172	59	CAROLINO 164	Chad	AUS	9.3	10.6	10.0
MC25	60	NORIN 11	Japan	TEJ	9.5	10.5	10.0
MC50	61	Chun 118-33	China	IND	10.0	10.0	10.0
MC55	62	GUYANE 1	Chad	IND	10.2	9.8	10.0
MC119	63	TAINUNG 45	Taiwan	IND	8.3	11.7	10.0
MC207	64	Pakkali	Philippines	ARO	12.0	8.0	10.0
MC208	65	THAVALU	Sri Lanka	AUS	9.5	10.7	10.1
MC53	66	BLUE STICK	Fiji	TEJ	9.3	10.9	10.1
MC41	67	Thang 10	Vietnam	IND	11.7	8.7	10.2
MC20	68	Italica Carolina	Poland	TEJ	10.6	9.8	10.2
MC213	69	WAB462-10-3-1	Cote D'Ivoire	TRJ	9.8	10.8	10.3
MC205	70	Magoti	Burundi	IND	10.2	10.5	10.3

MC48	71	Djimoron	Guinea	IND	12.2	8.8	10.5
MC113	72	Tranoeup Beykher	Cambodia	IND	10.8	10.3	10.6
MC154	73	Srav Prapay	Cambodia	IND	10.7	10.7	10.7
MC6	74	LEAH	United States	TRJ	9.7	11.8	10.8
Huazhan	75	Huazhan	China	IND	12.0	9.5	10.8
MC169	76	Tia Bura	Indonesia	TRJ	9.9	11.7	10.8
MC155	77	Nang Bang Bentre	Vietnam	AUS	11.0	10.7	10.9
MC63	78	MOROBEREKAN	Guinea	TRJ	10.4	11.5	11.0
MC18	79	81B/25	Suriname	IND	9.3	12.7	11.0
MC65	80	IR 9660-48-1-1-2	Philippines	IND	11.5	10.6	11.0
MC111	81	99216	India	AUS	9.3	12.8	11.1
MC104	82	WC 4443	Bolivia	TRJ	7.7	14.7	11.2
MC153	83	Karayal	Sri Lanka	AUS	11.0	11.3	11.2
MC160	84	Santhi 990	Pakistan	Admix(AUS-IND-WD)	9.5	12.8	11.2
MC52	85	Manga Kely 694	Madagascar	Admix(AUS-IND)	13.2	9.4	11.3
Diula	86	Diula	India	IND	9.8	12.8	11.3
Shu Hui498	87	Shu Hui498	China	IND	9.4	13.2	11.3
MC24	88	Safut Khosha	Afghanistan	AUS	9.0	13.7	11.3
MC73	89	BHIM DHAN	Nepal	Admix(TEJ-ARO-TRJ)	10.8	12.0	11.4
MC151	90	Gallawa	Sri Lanka	AUS	11.2	11.7	11.4
MC167	91	Romeno	Portugal	TEJ	7.8	15.0	11.4
MC97	92	Dular	India	AUS	11.7	11.2	11.4
MC26	93	R 75	Senegal	TRJ	9.0	14.0	11.5
MC17	94	SHIMIZU MOCHI	Japan	TEJ	10.7	12.4	11.5
9311	95	9311	China	IND	10.6	12.6	11.6
MC42	96	Sipirasikkam	Indonesia	TRJ	14.2	9.0	11.6
MC57	97	INIAP 7	Ecuador	IND	9.8	13.3	11.6
MC81	98	Ragasu	Taiwan	Admix(TEJ-TRJ)	10.2	13.0	11.6
MC14	99	BERLIN	Costa Rica	IND	8.3	15.0	11.7
MC114	100	10340	Italy	IND	12.0	11.3	11.7
MC163	101	Jyanak	Bhutan	Admix(TEJ-TRJ-ARO)	10.0	13.3	11.7
MC83	102	Tamanishiki	Japan	TEJ	10.0	13.5	11.8
MC166	103	Spin Mere	Afghanistan	AUS	9.3	14.2	11.8
MC201	104	SOC NAU	Vietnam	IND	10.0	13.7	11.8
MC3	105	E B Gopher	United States	TRJ	8.8	14.8	11.8
MC38	106	Chacareiro Uruguay	Uruguay	TEJ	10.3	13.3	11.8
MC59	107	Red	Pakistan	Admix(AUS-ARO-IND)	10.3	13.3	11.8

MC37	108	Won Son Zo No. 11	Korea	IND	13.8	9.9	11.8
MC152	109	Ittikulama	Sri Lanka	AUS	12.2	11.7	11.9
MC27	110	UZ ROSZ M38	Uzbekistan	TEJ	12.8	11.0	11.9
MC110	111	Hsin Hsing Pai Ku	Taiwan	IND	11.8	12.0	11.9
MC117	112	TD 70	Thailand	IND	12.8	11.0	11.9
MC115	113	AKP 4	India	IND	9.5	14.4	12.0
MC16	114	Sel. No. 388	Uruguay	Admix(TEJ-TRJ)	12.3	11.7	12.0
MC80	115	Kin Shan Zim	China	IND	11.5	12.5	12.0
MC168	116	AMANE	Sri Lanka	IND	12.0	12.0	12.0
MC74	117	RP2151-173-1-8	India	IND	10.9	13.3	12.1
MC76	118	ECIA76-S89-1	Cuba	IND	11.7	12.5	12.1
MC82	119	Tobura	Taiwan	Admix(TEJ-TRJ)	12.3	12.2	12.3
MC84	120	Yong Chal Byo	Korea_ South	Admix(TEJ-TRJ)	12.5	12.0	12.3
MC102	121	R 67	Senegal	TRJ	11.2	13.3	12.3
MC159	122	DNJ 121	Bangladesh	AUS	11.7	13.2	12.4
MC93	123	Juppa	Nepal	IND	11.8	13.0	12.4
MC29	124	IARI 6626	India	AUS	11.3	13.7	12.5
MC58	125	Onu B	Zaire	TRJ	15.7	9.3	12.5
MC70	126	PHUDUGEY	Bhutan	AUS	11.2	13.8	12.5
MC99	127	2	Afghanistan	ARO	11.7	13.3	12.5
MC112	128	Shui Ya Jien	Hong Kong	IND	12.0	13.0	12.5
MC7	129	Quinimpol	Philippines	TRJ	16.4	9.6	13.0
MC54	130	Nam Dawk Mai	Thailand	IND	14.8	11.3	13.1
MC15	131	British Honduras Creole	Belize	TRJ	12.7	13.5	13.1
MC47	132	K8C-263-3	Suriname	IND	12.7	13.5	13.1
MC72	133	Ak Tokhum	Azerbaijan	ARO	13.5	12.7	13.1
MC89	134	Niwahutaw Mochi	Japan	TEJ	12.6	13.7	13.1
MC194	135	Sereno	Jamaica	IND	9.9	16.4	13.1
MC67	136	Jumli dhan	Nepal	Admix(TEJ-ARO-TRJ)	12.8	13.5	13.2
MC85	137	Grassy	Haiti	TRJ	13.5	12.8	13.2
MC31	138	Khao Luang	Laos	TRJ	12.0	14.5	13.3
MC66	139	Bakiella 1	Sri Lanka	IND	6.3	20.2	13.3
MC90	140	6360	Turkey	TEJ	13.7	13.2	13.4
MC171	141	P 35	India	AUS	10.7	16.2	13.4
MC106	142	IARI 6621	India	AUS	8.2	18.7	13.4
MC77	143	WC 3532	Peru	TRJ	15.0	12.0	13.5
MC92	144	Buphopa	Myanmar	TRJ	11.4	15.7	13.5

MC116	145	SORNAVARI	Mali	AUS	10.2	17.0	13.6
MC10	146	Ao Chiu 2 Hao	China	IND	12.3	15.0	13.7
MC144	147	4484	China	IND	12.0	15.3	13.7
MC91	148	Somewake	Japan	TEJ	14.2	13.2	13.7
MC206	149	Pa Boup	Sierra Leone	AUS	13.4	14.0	13.7
MC78	150	GPNO 1106	Guatemala	TRJ	14.2	13.3	13.8
MC103	151	LUSITANO	Portugal	TEJ	10.8	16.7	13.8
MC143	152	KECHENGNUO NO. 4	China	IND	12.5	15.0	13.8
MC199	153	Coppocina	Bulgaria	TRJ	15.0	12.5	13.8
MC108	154	Gazan	Afghanistan	TEJ	10.4	17.3	13.8
MC161	155	UZ ROS 7-13	Uzbekistan	AUS	13.9	13.8	13.8
MC107	156	Mitak	Indonesia	TRJ	14.5	13.3	13.9
MC210	157	Krachek Chap	Indochina	IND	13.0	15.0	14.0
MC121	158	Sapundali Local	India	IND	14.8	13.4	14.1
MC46	159	PATNAI 6	Myanmar	AUS	13.7	14.7	14.2
MC8	160	TAICHU MOCHI 59	Taiwan	TRJ	13.8	15.0	14.4
MC101	161	CSORNUJ	Hungary	TEJ	14.7	14.3	14.5
MC131	162	Shimla Early	Iraq	Admix(AUS-IND)	14.3	14.8	14.6
MC95	163	TAINO 38	Taiwan	Admix(TRJ-AUS-IND-TEJ)	15.0	15.0	15.0
MC100	164	Vary Tarva Osla	Portugal	Admix(TEJ-TRJ)	12.8	17.2	15.0
MC62	165	IR 4482-5-3-9-5	Philippines	IND	13.6	16.7	15.1
MC11	166	Criollo Chivacoa 2	Venezuela	TRJ	12.3	18.0	15.2
MC130	167	CO 13	India	IND	16.0	14.3	15.2
MC105	168	IR 238	Philippines	IND	14.2	16.3	15.3
MC138	169	KAUKKYI ANI	Myanmar	TRJ	15.0	15.5	15.3
MC49	170	Anandi	India	IND	13.2	17.4	15.3
MC149	171	A 5	Japan	TEJ	14.4	16.4	15.4
MC9	172	WC 2811	Micronesia	TRJ	11.7	19.2	15.4
MC88	173	Pan Ju	China	IND	12.7	18.3	15.5
MC135	174	UZ ROS 59	Uzbekistan	IND	17.3	13.8	15.6
MC173	175	ASWINA 330	Bangladesh	AUS	17.8	13.4	15.6
MC64	176	KUBANETS 508	Russian Federation	TEJ	14.3	17.5	15.9
MC87	177	Kao Chio Lin Chou	Taiwan	IND	18.3	13.7	16.0
MC12	178	Bombilla	Spain	TEJ	20.7	11.7	16.2
MC96	179	17-9-4	Mexico	IND	16.0	16.6	16.3
MC214	180	cypress	United States	TRJ	17.0	16.0	16.5
MC61	181	BKN 6987-68-14	Thailand	IND	16.7	16.4	16.5

MC69	182	TCHAMPA	Iran	AUS	15.2	19.0	17.1
MC145	183	4595	China	IND	20.0	14.8	17.4
MC134	184	LA PLATA GENA F.A.	Argentina	AUS	21.2	13.8	17.5
MC126	185	B805D-MR-16-8-3	Indonesia	IND	16.2	19.0	17.6
MC147	186	CHUNJIANGZAO NO. 1	China	TEJ	16.0	19.2	17.6
MC148	187	Egyptian Wild Type	Turkey	TEJ	17.6	18.0	17.8
MC209	188	WC 10253	Uncertain	TRJ	16.0	20.0	18.0
MC123	189	Bombon	Spain	TEJ	18.7	17.5	18.1
MC128	190	Saraya	Fiji	AUS	17.7	18.5	18.1
MC170	191	Padi Tarab Arab	Malaysia	TRJ	20.2	16.3	18.3
MC150	192	C.B. II	Japan	AUS	15.8	20.8	18.3
MC125	193	79	Guyana	ARO	20.3	16.5	18.4
MC124	194	Dara	Indonesia	AUS	19.3	17.7	18.5
MC136	195	Gasym Hany	Azerbaijan	ARO	19.7	17.3	18.5
MC127	196	TONO BREA 439	Dominican Republic	IND	18.7	18.8	18.7
MC140	197	CNTLR80076-44-1-1-1	Thailand	IND	17.5	20.8	19.2
MC146	198	YOU-I B	China	IND	19.2	19.2	19.2
MC122	199	Tauli	Nepal	AUS	24.2	14.4	19.3
MC13	200	Secano do Brazil	El Salvador	TRJ	15.7	23.3	19.5
MC139	201	Celiaj	Azerbaijan	TEJ	16.7	22.3	19.5
MC132	202	Montakcl	Egypt	AUS	19.3	20.0	19.7
MC137	203	DARMALI	Nepal	Admix(TEJ-TRJ-AUS-ARO)	19.5	20.2	19.9
MC142	204	CM1_ HAIPONG	Vietnam	IND	14.7	25.2	19.9
MC141	205	IR 58614-B-B-8-2	Philippines	IND	20.2	19.8	20.0
MC71	206	WIR 3039	Tajikistan	TEJ	20.3	20.8	20.5
MC129	207	Botika S/R	Zaire	TRJ	21.5	22.0	21.8

Supplementary Table 2. Allelism test of *M. graminicola* -resistance genes between resistant varieties.

Population	Total plants	Resistant plants	Susceptible plants	Theoretical ratio (R:S)	χ^2	*P*
F_2 (SL 22-620×ZH11)	160	160	0	1:0	-	-
F_2 (HKG 98×ZH11)	297	297	0	1:0	-	-
F_2 (ZH11×Toga)	176	160	16	15:1	2.42	> 0.05
F_2 (HKG 98×Toga)	197	182	15	15:1	0.63	> 0.05

Supplementary Table 3. Potential palmitoylation sites in MG1 predicted with CSS-Palm 4.0.

ID	Position	Peptide	Score	Cutoff
Mg1	12	VDTLVGSCINKLQAI	32.13	3.717
Mg1	521	SLGTNTMCKVRRISV	4.231	3.717
Mg1	793	KYVLLIDCKSCVHLP	5.948	3.717

Supplementary Table 4. List of MG1-interacting candidates obtained from Y2H initial screening.

Candidate	Annotation	Gene ID	Length obtained	Number of times
1	serine protease inhibitor family	LOC_Os12g25090	Full	1
2	phosphofructokinase	LOC_Os10g26570	Partial	1
3	methyltransferase domain containing protein	LOC_Os04g42870	Partial	1
4	elongation factor Tu, putative	LOC_Os03g08020	Partial	1
5	GRAM domain containing protein	LOC_Os03g08860	Partial	1
6	dienelactone hydrolase family protein	LOC_Os01g34700	Partial	1
7	BTB domain with H family conserved sequence	LOC_Os04g20920	Partial	1
8	annexin	LOC_Os02g51750	Partial	1
9	WD domain, G-beta repeat domain containing protein	LOC_Os02g04320	Partial	1
10	cytoplasmic ribulose-phosphate 3-epimerase	LOC_Os09g32810	Partial	>15

Supplementary Table 5. Primers used in this study.

Name	Forward (5'-3')	Reverse (5'-3')	Utilization
FJ 5	TATATATGCTGTGGGGGTTC	CCCTTAGTGCCACATTAAAA	For physical mapping
FJ 6	GGTACGGTGGTGCTATATGT	GATTGATCCACCATCTTTCC	For physical mapping
CR 2	ATTTGCCAAACAAACCCAAG	TCCTTAGGGTTTGGAATTACG	For physical mapping
CR 8	GTCTTCCGTGGCGTAAATGT	TGAGTCAAGGGTGATCATGG	For physical mapping
WXM6	GTCACACCATTTGTAGGCTC	TTTGGAGGGAAGGAGTATGC	For physical mapping
CR 4	CAAAGGTTGAAGACGAGAGG	CGGAGGGAGTAACTATGTAGTG	For physical mapping
CR 7	GCTAAAGTTGAACTGCGG	CAACCGTTTTTGGGCACAA	For physical mapping
TJ146-7	AAGCTGTCCTTTTTGTCAGA	CTTGAAACCTCAAAGAATGG	For physical mapping
WXM18	CCTATCCAGCTATTCTTTCAG	GGGAATGAAGTCTTTCAGG	For physical mapping
WXM37	CACATGCACTCATCTCTTGC	GAAAACAGAGACAAATCTGGTC	For physical mapping
MH14	CTCCCAAAACAAGTACCTTC	CTTGTTTGTTGTCTAGTGTGG	For physical mapping
CR 11	ACGAGGGAAGCTCAAGTT	TACCACCAGCTCGCTTTATC	For physical mapping
CR 16	CGATCCATCGTCATCACT	CATGCATGGGAGCATCCAAA	For physical mapping
CR 17	AACCAGACAAGTGACTGG	TCGAGCGGAGATATTGTG	For physical mapping
CR 20	TGCTCCTAGACCACTAAGGT	ACTGCCCAAATCTTCGAG	For physical mapping
CR 28	TGTCTGCCCCAATTTGAG	TAGTGTACATACCTTGCGC	For physical mapping
188-3	CAATTCTCGGAATGCCCCTA	GTGATTATGACATCCATCCCCT	For physical mapping
TJ146-3	GGGGTGCAGTTACACTTTAG	TCCGAAGGAAAGTTTTTATG	For physical mapping
11-20M	ATTTGCCAAACAAACCCAAG	TCCTTAGGGTTTGGAATTACG	For physical mapping
11-25M	GTCTTCCGTGGCGTAAATGT	TGAGTCAAGGGTGATCATGG	For physical mapping
WXM15	AGAGACAGCATAAGAAGC	GGACCTTCAGTTGTAAGTTC	For physical mapping
TJ146-11	CCCTCCATTTCACAATAAGA	GATAATTGTATGGTCTACTCTCTCC	For physical mapping
WXM35	CATTTCACCAGCTTGAGAATGAGC	GCTAATTGAACTGAACCCTACCAG	For physical mapping
TJ146-14	TTGCTTTACCTCATGAAATG	CCGGCTATGTCATTAAAATC	For physical mapping
11-27M	TCCCGCTATTCGATGTTACC	ACCAATGATTGCTTCTCCAA	For physical mapping
TJ146-5	AGCTTTAGGCCCTTCATAGT	GCTTCCAAGTGAAGATATGG	For physical mapping
WXM39	CAGGAAGTTACTTGGTCGAG	CGCTCATTAGCATCACGGTTAG	For physical mapping
MH13	TAGGACCTCCATAGACCCATG	CTAAGGACTTAATGCCCAG	For physical mapping
MH15	CTCCACACATATGTCATCAG	GATTGATGTAGGATTTGAGC	For physical mapping
MH4	ACTTATTCTTGGCAGTCGG	CCTCATAAATCAACTGATGG	For physical mapping
OsActin	TCCTTCGTCTCGACCTTGCTGG	CACCACTGAGAACGATGTTGCCATA	For RT-PCR
OsEXPNAR	AGAAGGCCATGAACATCCAGTGC	AAGCTCACGCTGCTTTGCTACC	For qRT-PCR
qC4	GATTATTTGGTTGGATCATGTGCC	CCTTGCCTCAGCATCACTAATAC	For qRT-PCR
qC5	CGAATTATGGATGATCAAG	TGCAGCATGCAACGGAATTC	For qRT-PCR
qC61	GCTCGAAATGTTGATCTTCAA	GGCAGTTCACAAGTTGCAACTCAT	For qRT-PCR

qC62	GCTCGAATGGTTGGTCATTGG	CCCACAAGTGTCAACCTCGTCA	For qRT-PCR
qC9	AAGGCCATGGAATTCCCCTGTTG	TTCTAGAGATTTGGTGAGGC	For qRT-PCR
OsPR10	GCGGGAGTCGGGAATACATACAAG	TCCAGCACCTCTGACTTTAGCACG	For qRT-PCR
OsPR1a	GCTGTACTGTCAGCCGTATTTGC	ACCATGCATGTAACCACGAAGGAC	For qRT-PCR
OsWRKY45	AGCAATCGTCCGGGAATTCG	TGCACAGCTGGTCGTACTTG	For qRT-PCR
OsCRK5	TCAGGAGGTTGTTCTCGTAG	TGCACTTTGCTCGTTCTCCTC	For qRT-PCR
q45050	TCTTCACTGTGGGTCCCAAGG	CTGAACTTGTGGCCGTTTAC	For qRT-PCR
SalI QC GC61	ttgcatgcctgcaggtcgacTACACTTTGGGCCTCACATTGCTC	gtacccggggatcctctagaTAGGCGGAGTTACCTACACACCT	For complementary assay
G C61	ggggatcctctagagtcgacATGGCAACAATAGTAGACAC	gagccctggcatgcctgcagTCACAGCCACTCGATGACCT	For complementary assay
C61pro-SalI	caggtcgacGTACACTTTGGGCCTCACATTG	cgggaattcCCGACAAACTCAAAAGGGGTAATC	For complementary assay
C3g-1	taGGTCTCCAACAAAATCCACgttttagagctagaa	cgGGTCTCATGTTGGGTTCTAtgcaccagccggg	For complementary assay
C3g-2	taGGTCTCCAGTGCAATCACTgttttagagctagaa	cgGGTCTCACACTAGGCAAACtgcaccagccggg	For complementary assay
C4C5g-1	taGGTCTCCGATGCTGAGGCAgttttagagctagaa	cgGGTCTCACATCACTAATACtgcaccagccggg	For complementary assay
C4C5g-2	taGGTCTCCTCATCGTCATCAgttttagagctagaa	cgGGTCTCAATGAAGGATGATtgcaccagccggg	For complementary assay
C61g-1	taGGTCTCCCAGACATCATAAgttttagagctagaa	cgGGTCTCATCTGCGGTTGAGtgcaccagccggg	For complementary assay
C61g-2	taGGTCTCCTACAAACTTCTCgttttagagctagaa	cgGGTCTCATGTAAAACACCTtgcaccagccggg	For complementary assay
C62g-1	taGGTCTCCCACACAACATAAgttttagagctagaa	cgGGTCTCATGTGCGGCTGAGtgcaccagccggg	For complementary assay
C62g-2	taGGTCTCCTTTGTGTCTCCAgttttagagctagaa	cgGGTCTCACAAACCCAGGCAtgcaccagccggg	For complementary assay
C9g-1	taGGTCTCCATTTGGTGAGGCgttttagagctagaa	cgGGTCTCAAAATCTCTAGAAtgcaccagccggg	For complementary assay
C9g-2	taGGTCTCCAGCAAACATGCAgttttagagctagaa	cgGGTCTCATGCTGAAGCTTCtgcaccagccggg	For complementary assay
3C4-1F	AGCTGCGCGTGCAAGATTGTCCAA		3’ RACE 1st round RT-PCR
3C4-2F	CGGTTGTTTGATGCAAGGTATGTG		3’ RACE 2st round RT-PCR
3C5-1F	AATTTGCTGGCCTCAGGGCGA		3’ RACE 1st round RT-PCR
3C5-2F	AGAGCTGCGCATGCAAGATTG		3’ RACE 2st round RT-PCR
3C61-1	GAGACACAAGATACTTGAAGACG		3’ RACE 1st round RT-PCR
3C61-2	TCTTCACTGTGGGTCCCAAGG		3’ RACE 2st round RT-PCR
3C62-1	ACGAGGTTGACACTTGTGGGCT		3’ RACE 1st round RT-PCR
3C62-2	CTGCGTGTAAATGTCTGCCCC		3’ RACE 2st round RT-PCR
3C9-1	CTCCTTGGAGTAGTACATATTC		3’ RACE 1st round RT-PCR
3C9-2	CTTGCCTTCTGTGAAATCCGTG		3’ RACE 2st round RT-PCR
5C4-1R	CATGATGAACTTCCATAATTCG		5’ RACE RT
5C4-2R	GATGACGATGAAGGATGATCAG		5’ RACE 1st round RT-PCR
5C4-3R	GGCACATGATCCAACCAAATAATC		5’ RACE 2st round RT-PCR
5C5-1R	CATCTTGATCATCCATAATTCG		5’ RACE RT
5C5-2R	CAGTGCAAGCAATTGTCTTCC		5’ RACE 1st round RT-PCR
5C5-3R	AGCACATGATCCAACCAAAGAATC		5’ RACE 2st round RT-PCR
5C61-1R	GCATCCTGAAGAGAAGATCTT		5’ RACE RT
5C61-2R	CCCAGAGAAGTTTGTAAAACACC		5’ RACE 1st round RT-PCR

5C61-3R	ACCACTTCTCTGCAAGCACGTATG	5’ RACE 2st round RT-PCR
5C62-1R	CTCTGCAAGCATGTACGACC	5’ RACE RT
5C62-2R	CATTATGTTGTGTGCGGCTG	5’ RACE 1st round RT-PCR
5C62-3R	CTTATGAGATCTGTTCTTCGC	5’ RACE 2st round RT-PCR
5C9-1R	AGCACGAGAGAAGTGAAAGCC	5’ RACE RT
5C9-2R	GTCATCAGCGTCATACATAGC	5’ RACE 1st round RT-PCR
5C9-3R	CGCATGATCCAATCAAAGAATCCAG	5’ RACE 2st round RT-PCR
Qc	CCAGTGAGCAGAGTGACGAGGACTCGAGCTCAAGCC CCCCCCCCCCCCCCCC	Linker primer
Qo	CCAGTGAGCAGAGTGACG	Linker primer
Qi	GAGGACTCGAGCTCAAGC	Linker primer
